# The projected impact of geographic targeting of oral cholera vaccination in sub-Saharan Africa: A modeling study

**DOI:** 10.1371/journal.pmed.1003003

**Published:** 2019-12-11

**Authors:** Elizabeth C. Lee, Andrew S. Azman, Joshua Kaminsky, Sean M. Moore, Heather S. McKay, Justin Lessler

**Affiliations:** 1 Department of Epidemiology, Johns Hopkins Bloomberg School of Public Health, Baltimore, Maryland, United States of America; 2 Department of Biological Sciences, University of Notre Dame, Notre Dame, Indiana, United States of America; 3 Eck Institute for Global Health, University of Notre Dame, Notre Dame, Indiana, United States of America; The National Institute for Public Health and the Environment, NETHERLANDS

## Abstract

**Background:**

Cholera causes an estimated 100,000 deaths annually worldwide, with the majority of burden reported in sub-Saharan Africa. In May 2018, the World Health Assembly committed to reducing worldwide cholera deaths by 90% by 2030. Oral cholera vaccine (OCV) plays a key role in reducing the near-term risk of cholera, although global supplies are limited. Characterizing the potential impact and cost-effectiveness of mass OCV deployment strategies is critical for setting expectations and developing cholera control plans that maximize the chances of success.

**Methods and findings:**

We compared the projected impacts of vaccination campaigns across sub-Saharan Africa from 2018 through 2030 when targeting geographically according to historical cholera burden and risk factors. We assessed the number of averted cases, deaths, and disability-adjusted life years and the cost-effectiveness of these campaigns with models that accounted for direct and indirect vaccine effects and population projections over time. Under current vaccine supply projections, an approach optimized to targeting by historical burden is projected to avert 828,971 (95% CI 803,370–859,980) cases (equivalent to 34.0% of projected cases; 95% CI 33.2%–34.8%). An approach that balances logistical feasibility with targeting historical burden is projected to avert 617,424 (95% CI 599,150–643,891) cases. In contrast, approaches optimized for targeting locations with limited access to water and sanitation are projected to avert 273,939 (95% CI 270,319–277,002) and 109,817 (95% CI 103,735–114,110) cases, respectively. We find that the most logistically feasible targeting strategy costs US$1,843 (95% CI 1,328–14,312) per DALY averted during this period and that effective geographic targeting of OCV campaigns can have a greater impact on cost-effectiveness than improvements to vaccine efficacy and moderate increases in coverage. Although our modeling approach does not project annual changes in baseline cholera risk or directly incorporate immunity from natural cholera infection, our estimates of the relative performance of different vaccination strategies should be robust to these factors.

**Conclusions:**

Our study suggests that geographic targeting substantially improves the cost-effectiveness and impact of oral cholera vaccination campaigns. Districts with the poorest access to improved water and sanitation are not the same as districts with the greatest historical cholera incidence. While OCV campaigns can improve cholera control in the near term, without rapid progress in developing water and sanitation services or dramatic increases in OCV supply, our results suggest that vaccine use alone is unlikely to allow us to achieve the 2030 goal.

## Abstract 2

In this modeling study, Elizabeth Lee and colleagues estimate the effectiveness of different strategies for targeting oral cholera vaccination in sub-Saharan Africa.

## Author summary

**Why was this study done?:**

- Cholera is a waterborne infectious disease with substantial disease burden in sub-Saharan Africa.
- Oral cholera vaccines are safe and effective, but they have not often been considered for routine, preventive use because global supplies are limited.
- A recent World Health Assembly resolution declared a commitment to reduce cholera mortality by 90% by 2030, raising the importance of understanding how geographic targeting of routine mass cholera vaccination campaigns can maximize the health impact and cost-effectiveness of these programs.

**What did the researchers do and find?:**

- We developed a phenomenological model that examines the projected health impacts and cost-effectiveness for different vaccination campaign targeting strategies across sub-Saharan Africa from 2018 to 2030.
- The model leverages previous high-resolution estimates of cholera incidence and accounts for direct and indirect vaccine effects and population changes over time.
- The model suggests that targeting vaccination to districts based on historical cholera burden is significantly more effective than targeting districts based on indicators of access to water and sanitation infrastructure.
- We found that targeting vaccination campaigns geographically improved cost-effectiveness more than increasing vaccine supply or vaccination coverage.

**What do these findings mean?:**

- Effective geographic targeting of mass cholera vaccination campaigns optimizes the use of limited global cholera vaccine supplies.
- Targeting districts with the highest historical cholera risk leads to greater reductions in cholera incidence than targeting districts with poorest access to improved water and sanitation infrastructure.
- Despite a substantial projected health impact, mass vaccination campaigns cannot achieve the ambitious 2030 goal without large increases in access to improved water and sanitation.

## Introduction

Cholera remains a significant global public health threat, causing more than 100,000 deaths per year globally, with sub-Saharan Africa bearing the majority of the burden [1–3]. In May 2018, the 71st World Health Assembly adopted a resolution aimed at reducing global cholera deaths by 90% by 2030 [4]. Achieving major reductions in morbidity and mortality in sub-Saharan Africa is essential to reaching this goal.

Due to successful campaigns conducted in over 15 countries between 2013 and 2018 [5], countries that regularly experience cholera are beginning to integrate killed oral cholera vaccine (OCV) into regular public health activities, as recommended by the Global Task Force on Cholera Control (GTFCC) “roadmap to 2030” [6] and general WHO guidance [7]. These vaccines have 49%–67% efficacy in protecting vaccine recipients against cholera infection for up to 5 years [8], thus presenting an important near-term solution to rapidly reducing cholera risk while long-term improvements to safely managed and sustainable water and sanitation services are made. Moreover, killed whole-cell cholera vaccines are known to be safe. Trials of vaccines currently included in global vaccine stockpiles have not found evidence for severe vaccine-related adverse events in non-pregnant or pregnant populations [9,10].

Nevertheless, OCV presents several challenges to traditional approaches to vaccine deployment. OCV does not provide lifelong immunity; while the length of protection is uncertain, it is thought to wane significantly after 5 years [8]. Further, the vaccine appears to be half as protective in children under 5 years old [8]. Together, these factors suggest that inclusion of OCV in a childhood vaccination schedule would have limited impact. Similarly, the high degree of clustering of cholera risk, both geographically and demographically [11,12], suggests that large-scale (e.g., country-wide) vaccination campaigns may be inefficient.

The limited supply of OCV further complicates its integration into routine cholera control activities. In 2018, 23 million doses of OCV were produced globally (enough to provide the recommended 2-dose course to 11.5 million people) [11]. This is only a fraction of what would be needed to cover the 87.2 million people living in high-risk areas of sub-Saharan Africa alone [11]. Consequently, efficient strategies are needed if the limited vaccine supply is to play a significant role in cholera control.

While previous work has examined the impact of targeting OCV to specific age groups [13], an alternate strategy for efficient OCV use is to target vaccines geographically to high-risk areas. Geographic targeting may be particularly important in sub-Saharan Africa, where there is great heterogeneity in cholera dynamics; cholera has an endemic presence in countries like the Democratic Republic of the Congo, Mozambique, and Nigeria, yet causes only sporadic epidemics, separated by multiple years of inactivity, in other places. While cholera outbreaks are often catalyzed by conflict and climate-related events, their magnitude and duration are sustained by limited access to improved water and sanitation, key cholera risk factors.

In this work, we use simulation studies to explore the impact of different approaches to conducting geographically targeted OCV campaigns from 2018 to 2030 in sub-Saharan Africa. We quantify the impact of targeted strategies over untargeted OCV use and compare targeting according to historical cholera burden to that according to cholera risk factors. The ultimate aim of these analyses is to provide guidance on how this critical cholera control tool may be used efficiently to accomplish global cholera control goals.

## Methods

### Epidemiologic and demographic data sources

Our methods for mapping cholera incidence have been previously described [11]. Briefly, our estimates of cholera incidence were based on suspected and clinically confirmed cholera case reports from 2010 to 2016 obtained from multiple sources, including the World Health Organization (WHO), Médecins Sans Frontières, ProMED, situation reports from ReliefWeb and other websites, several ministries of health, and the scientific literature [3,11]. These cholera reports were combined with ecological risk factors such as access to improved drinking water and sanitation and distance to the nearest major body of water to estimate average annual cholera incidence rates at 20 km × 20 km grid resolution in a Bayesian modeling framework [3,11,14,15]. We did not obtain water and sanitation data for Botswana, Djibouti, and Eritrea, and these countries were excluded from our analyses. Summaries of all cholera datasets, including 20 km × 20 km resolution estimates of cholera cases and incidence, and instructions for requesting access are available at http://www.iddynamics.jhsph.edu/projects/cholera-dynamics.

Population estimates were derived from WorldPop’s population density rasters for Africa [16,17]. The population for each 1 km × 1 km grid cell was fit independently to population estimates and projections from WorldPop for 2000, 2005, 2010, 2015, and 2020 using a log-linear model. Annual population estimates from 2018 through 2030 were then projected for each cell independently using the fit log-linear rate of population change.

### Model simulation

We chose to use a phenomenological model to deal with cholera incidence and the impact of immunity on transmission, as classical mechanistic transmission models (e.g., SIR models) tend to perform poorly at coarse time resolutions and overestimate case counts. In the context of a spatial analysis for all districts (International Organization for Standardization second-level, sub-national administrative unit) in sub-Saharan Africa, such a transmission model would require many assumptions about heterogeneity in cholera disease dynamics that are not currently supported by data.

Our models were simulated in a deterministic manner at a 5 km × 5 km grid resolution. We chose this grid resolution as a balance between computational feasibility and accurate district-level population sizes (as summed across corresponding grid cells); 20 km × 20 km grid resolution provided highly inaccurate district population estimates, which consequently affected our vaccine targeting exercise. The input cholera incidence rate estimates were disaggregated from a 20 km × 20 km to a 5 km × 5 km grid (5 km × 5 km rates were the same as the rate for the corresponding 20 km × 20 km cell), and the WorldPop projections for a given year were aggregated from a 1 km × 1 km to a 5 km × 5 km grid.

We summarized the results across individual model simulations with the mean and a confidence interval calculated from 2.5 and 97.5 percentile results for a given modeling scenario. The uncertainty expressed in the reported confidence intervals reflects the statistical uncertainty captured in the posterior distribution of mean annual incidence across sub-Saharan Africa [11]. We used the same fixed set of 1,000 posterior draws of mean annual incidence for all years, vaccine deployment strategies, and sensitivity analyses.

### Vaccine properties

#### Campaign coverage

We conducted a review of published literature on post-OCV campaign vaccination coverage surveys and identified 7 studies related to 24 2-dose campaigns conducted globally from 2003 through 2016 (Table A in S1 Appendix) [18–26]. For each of the 7 studies, we resampled 2-dose (the standard vaccine regimen) coverage estimates 5,000 times from a Gaussian distribution with a mean equal to the estimated coverage at the campaign site and the variance derived from the associated 95% confidence intervals; for studies with multiple locations, we first drew a single location randomly and then sampled from a Gaussian distribution of coverage estimates for that location. We pooled these 35,000 draws across studies and used the median (68%) of the samples as the baseline coverage estimate for our model (Fig A in S1 Appendix). These estimates give studies equal weight regardless of the number of campaign locations and campaign site sample size.

#### Vaccine efficacy

We fit a log-linear decay function to 2-dose vaccine efficacy data reported 0 to 5 years after vaccination in a recent meta-analysis [8], and used the mean point estimates for each year as (direct) vaccine efficacy in our model. In this framework, the initial vaccine efficacy is 66%, declining to 0% at the end of the fifth year (Fig B in S1 Appendix). We modeled vaccination with only the full 2-dose regimen with no wastage.

#### Vaccine indirect effects

OCV has been shown to induce indirect protection across multiple settings [26–28]. We modeled indirect protection as a function of the vaccination coverage in a given grid cell using data from trials in India and Bangladesh [27,28]. Specifically, the phenomenological association between the relative reduction in incidence among unvaccinated (placebo) individuals and OCV coverage in their “neighborhood” was fit to a logistic function (Fig C in S1 Appendix). Under this model of indirect vaccine protection, individuals not protected by vaccine and residing in grid cells with 50% and 70% vaccination coverage experienced an 80% and near 100% reduction in cholera risk compared to no vaccination scenarios, respectively.

### Vaccine supply projections

Current global supplies of OCV are limited. In 2017, approximately 17 million bivalent killed whole-cell OCV doses of Shanchol (Shantha Biotech, Hyderabad, India) and Euvichol-Plus/Euvichol (Eubiologics, Seoul, Republic of Korea) were produced. Based on estimates from experts within the GTFCC and data from vaccine manufacturers in the first half of 2018, we assumed that global OCV supply would increase linearly from 23 million doses in 2018 to 59 million doses in 2030 (Fig E in S1 Appendix).

### Vaccination deployment strategies

**Fig 1:**
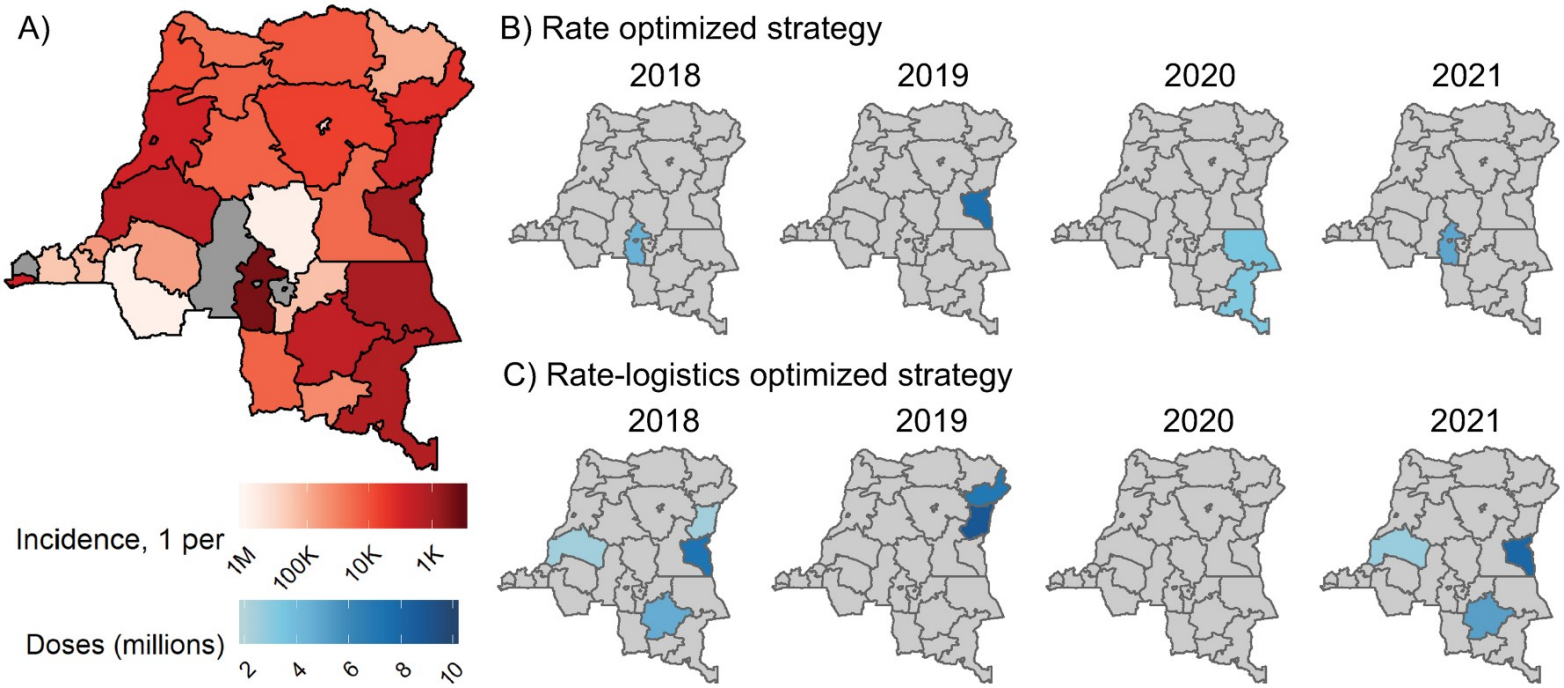
Demonstration of district-level vaccination deployment strategies in the Democratic Republic of the Congo (DRC). (A) Estimated annual incidence rate by district (International Organization for Standardization second-level, sub-national administrative unit) in 2018. Districts in grey had an annual incidence rate less than 1 per million people. Base maps were sourced from GADM (https://gadm.org). Vaccine allocations in the DRC by year according to the (B) rate-optimized and (C) rate-logistics-optimized strategies. Districts were targeted in a second consecutive year if the first year’s campaign did not have enough vaccine to cover the target population. Districts in grey were not targeted in DRC that year, and there were no districts targeted in the DRC in 2020 in the rate-logistics-optimized strategy.

We modeled 8 OCV deployment strategies: untargeted distribution, 4 historical-burden-based strategies (rate optimized, rate-logistics optimized, case optimized, and case-logistics optimized) (Fig 1), and 3 based on access to improved water and sanitation (water optimized, sanitation optimized, and water and sanitation [watsan] optimized), as defined by WHO/UNICEF Joint Monitoring Programme for Water Supply, Sanitation and Hygiene (JMP) indicators. For all targeted strategies, a given district was targeted fully (i.e., achieving 68% vaccination coverage) only once every 3 years, as suggested by WHO recommendations for OCV deployment [29]. Districts were ranked only once, and vaccination campaign targets changed annually according to parameters on vaccination campaign frequency and vaccine supply.

#### Untargeted distribution

OCV is distributed proportional to population throughout the study region (i.e., everyone has equal likelihood of receiving vaccine).

#### Rate optimized

We ranked all districts across countries in the study area by estimated cholera incidence rate (i.e., cases per unit population) and targeted them in decreasing order with vaccine until the annual global vaccine supply was depleted (Fig G in S1 Appendix).

#### Rate-logistics optimized

It may not be logistically feasible to target districts in multiple, potentially geographically nonadjacent countries at once. Thus, in the rate-logistics-optimized strategy, we ranked and selected countries according to the size of the population residing in high-risk districts, and within the selected countries, targeted vaccines to all high-risk districts. The highest risk districts were defined as those where cholera incidence exceeded a threshold of 1 case per 1,000 persons for at least 100,000 residents, or 10% of the population; subsequent tiers of high-risk districts employed incidence thresholds of 1 case per 5,000, 10,000, and 100,000 persons successively (Fig H in S1 Appendix) [3]. Within each incidence threshold tier, districts were ranked from highest to lowest by estimated cholera incidence rate (hence, rate-logistics). Some districts with high incidence rates may not meet the definition of high-risk district in this strategy because fewer than 100,000 people or less than 10% of the district population reside in cells achieving the given incidence threshold tier; this can lead to differences in targeting between the rate-optimized and rate-logistics-optimized strategies.

#### Case optimized and case-logistics optimized

These are the same as their rate-optimized counterparts, except districts were ranked according to raw cholera case numbers instead of estimated cholera incidence rates (S1 Appendix only).

#### Water optimized, sanitation optimized, and watsan optimized

We ordered districts across all countries according to their estimated coverage of access to improved water, improved sanitation, and improved watsan (S1 Appendix only), and then targeted them from worst to best coverage. District-level coverage was derived from previously modeled estimates of access to improved water and improved sanitation [14].

### Measuring public health impact and costs

We estimated the public health impact of conducting OCV campaigns from 2018 to 2030 as the number of cholera cases, deaths, and disability-adjusted life years (DALYs) averted over the period from 2018 to 2030 (see S1 Appendix).

We reviewed 4 cost surveys for mass OCV campaigns that reported the vaccine delivery and vaccine procurement costs per fully vaccinated person (i.e., receiving 2 vaccine doses) in non-refugee African settings [30–33] (Table B in S1 Appendix). One outlying data point with high vaccine purchase prices ($10 in 2009 US dollars [USD]) was excluded [33], as ongoing policy discussions suggest that future, preventive use of OCV in these settings will likely be available at lower prices. All costs were adjusted to 2017 USD according to the World Bank Consumer Price Index. The mean total cost per fully vaccinated person ($6.32), calculated as the sum of delivery and procurement costs, was used to calculate vaccination campaign costs.

Program costs were measured as the cost per DALY averted (2017 USD), and discount rates for health benefits and costs were set to 0% and 3%, respectively [34]. We calculate total costs per DALY averted for all combinations of the 3 cost estimates and 1,000 samples of averted DALYs (per model) in order to estimate 95% confidence intervals jointly from these 2 empirical distributions.

### Sensitivity analyses for model parameters

We performed 1-way sensitivity analyses of variability in vaccination deployment strategy, vaccine efficacy, indirect vaccine protection, vaccination campaign coverage, vaccine supply, net loss of vaccinated individuals due to migrations and deaths (i.e., population turnover rate), and the time between vaccination campaigns in a given location (i.e., vaccination campaign frequency; Table 1). Sensitivity parameters for direct vaccine efficacy were taken directly from the upper and lower 95% confidence interval bounds from a recent meta-analysis (Fig B in S1 Appendix) [8]. For indirect vaccine protection, we assumed, as a lower bound, that the vaccine conferred no indirect protection (Fig D in S1 Appendix). On the upper end, we assumed that individuals not directly protected by vaccine residing in grid cells with 30%, 50%, and 70% vaccination coverage experienced a respective 66%, 88%, and 97% reduction in cholera risk relative to a no vaccination scenario, according to a logistic model fit to published estimates (Fig D in S1 Appendix) [35]. Sensitivity parameters for vaccination coverage were taken from the 10th and 90th percentile resampled distribution of published coverage survey estimates from previous OCV campaigns (Fig A and Table A in S1 Appendix) [18–26]. Our sensitivity analysis for vaccine supply assumed, after 2019, either no growth or linear growth to 95 million doses in 2030; this upper limit vaccine supply may be achieved if new OCV production facilities open as planned (Fig F in S1 Appendix). We explored the sensitivity of our primary estimates to assumptions of the population turnover rate using data from 2017 UN World Population Prospects projections. The upper and lower bounds of population turnover rate were represented by the 5th and 95th percentiles of life expectancy for African countries in our study for 2018–2030, 56 and 70 years old, respectively [36].

**Table 1:** Parameters and references for vaccination campaign performance and costs for the primary model and sensitivity analyses.

Model parameter	Primary scenario	Sensitivity lower bound	Sensitivity upper bound
Vaccine efficacy (percent after 1–5 years) [8]	60, 52, 43, 32, 20	49, 44, 29, 4, 0	68, 59, 54, 52, 51
Indirect vaccine protection (percent at 10%, 30%, 50%, 70%, 90% coverage) [27,28,35]	0.09, 7, 84, 100, 100	0, 0, 0, 0, 0	34, 66, 88, 97, 99
Vaccination coverage (percent) (references in Table A in S1 Appendix)	68	50	84
Vaccine supply (2018–2030 in millions)	23–59	23–26	23–95
Population turnover rate (years^−1^) [36]	65	70	56
Vaccination campaign frequency	Every 3 years	Every 5 years	Every 2 years
Vaccine delivery cost per FVP (2017 USD)^1^ (references in Table B in S1 Appendix)	2.33	Not applicable	Not applicable
Vaccine procurement cost per FVP (2017 USD)^2^ (references in Table B in S1 Appendix)	5.49	Not applicable	Not applicable

^1^Vaccine delivery costs are related to program preparation, administration, and adverse events following immunization.

^2^Vaccine procurement costs are related to vaccine price, shipment, and storage.

FVP, fully vaccinated person; USD, US dollars.

### Projected cases averted due to vaccination campaigns

For our study period years of 2018–2030, we assumed that cholera incidence remained constant at the mean annual incidence rate observed from 2010–2016 in the absence of vaccination. Exploratory analyses using annual cholera reports to the WHO suggest that mean annual incidence may be an unbiased estimator for annual incidence at the country level, which suggests that this assumption about projected cholera incidence is valid in the expectation (Fig K in S1 Appendix), although uncertainty remains underestimated.

We assume that cholera vaccination is the only mechanism that confers immunity to cholera. The proportion of the population not protected by vaccine, and hence susceptible to cholera infection (“susceptibles”), in location *i* in year *t* is

$$S_{i,t}^{*}=\Pi_{k=1}^{t}\left(1-V_{i,k}f_{v}\left(t-k+1\right)p_{i,k,t}\right)$$

where *V*_*i*,*k*_ is the proportion of the population vaccinated in year *k* at location *i*. The function *f*_*v*_(*n*) represents the direct vaccine efficacy *n* years after vaccines were administered to the population. We modeled demographic changes in the population as

$$p_{i,k,t}=\left(N_{i,k}\left(1-\left(t-k\right)\mu \right)\right)/N_{i,t}$$

where *p*_*i*,*k*,*t*_ is the proportion of the population in location *i* from year *k* that is still present in year *t*. This proportion is calculated as a function of the population *N* in year *k* and location *i*, and the location’s net loss of vaccinated individuals due to migrations and deaths, *μ*. We assumed that the net loss of vaccinated individuals due to migrations and deaths (population turnover rate) was the same for all locations and tied roughly to median life expectancy across African countries in our data for 2018–2030 (65 years^−1^) [36]. The expected number of cholera cases at time *t* and location *i*, *Y*_*i*,*t*_, is then calculated as

$$Y_{i,t}=S_{i,t}\lambda_{i}N_{i,t}$$

where *S*_*i*,*t*_ is the susceptible proportion of the population after accounting for both direct and indirect vaccine effects, and λ_*i*_ is the projected baseline cholera incidence for location *i*. The total proportion of effective susceptibles *S*_*i*,*t*_ may be represented as

$$S_{i,t}=S_{i,t}^{*}\times g_{v}\left(S_{i,t}^{*}\right)$$

where *g*_*v*_ is a function that models the indirect effects of vaccination, as described in the Vaccine Properties section above (Fig C in S1 Appendix). To estimate the potential reductions in cases attributable to OCV use, we calculated

$$Y_{i,t}^{*}-Y_{i,t}$$

where $Y_{i,t}^{*}$ represents the counterfactual scenario where no vaccines were deployed.

## Results

Absent substantial changes to cholera prevention and control, or secular trends in incidence, we expect 2.44 (95% CI 2.41–2.48) million reported cholera cases from 2018 through 2030 in sub-Saharan Africa, with nearly 50% of these cases in just 4 countries—the Democratic Republic of the Congo, Nigeria, Somalia, and Sierra Leone. Given this clustering of disease burden, we examined practical deployment strategies targeting the highest risk districts across sub-Saharan Africa according to historical cholera burden (rate optimized and rate-logistics optimized) and to water and sanitation coverage (water optimized and sanitation optimized) and assessed the sensitivity of our results to different vaccine-related assumptions (with additional case-optimized, case-logistics-optimized, and watsan-optimized strategies considered in S1 Appendix).

**Fig 2:**
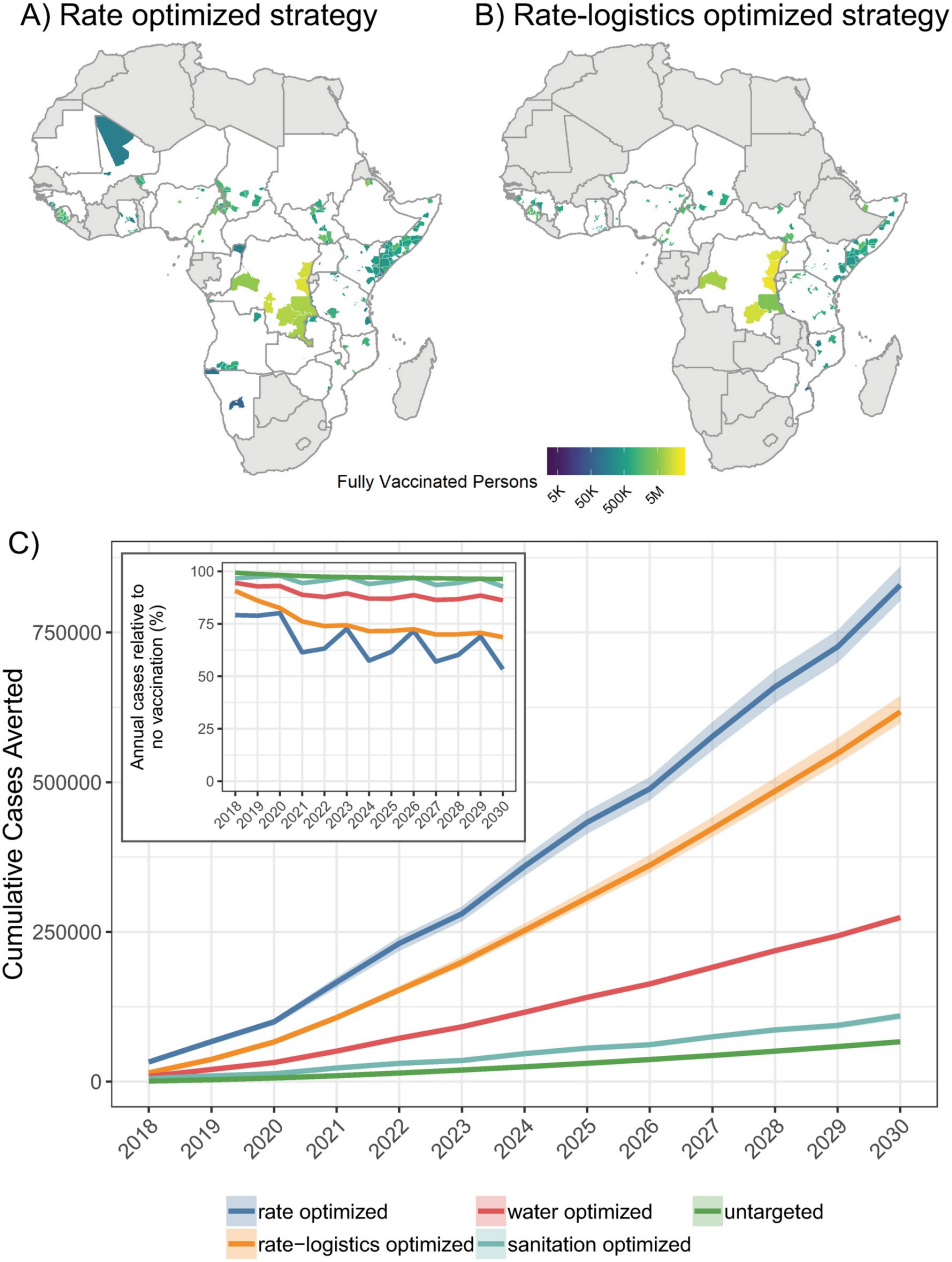
Health outcomes after vaccination under primary model assumptions. Cumulative number of fully vaccinated persons in sub-Saharan Africa as a result of campaigns from 2018 through 2030 according to the (A) rate-optimized and (B) rate-logistics-optimized vaccination deployment strategies. Countries in grey had no districts targeted by a given vaccination deployment strategy. Base maps were sourced from GADM (https://gadm.org). (C) Cumulative cases averted from mass oral cholera vaccination campaigns across 5 deployment strategies in sub-Saharan Africa from 2018 through 2030 (mean and 95% CI). The inset figure shows 1 minus the mean annual percentage of cholera cases averted in our models according to each deployment strategy.

When targeting districts ranked by expected cholera incidence rate (rate optimized), 34.0% (95% CI 33.2%–34.8%) of cases that would have otherwise occurred without vaccination from 2018 through 2030 were averted (Fig 2). This reduction translates to 828,971 (95% CI 803,370–859,980) cases, 31,958 (95% CI 31,503–33,011) deaths, and 746,749 (95% CI 736,607–762,273) DALYs averted after vaccination campaigns from 2018 through 2030 (Figs M–O and Table D in S1 Appendix). Due to our model assumption that baseline cholera risk will remain constant over the study period, the rate-optimized strategy represents the “best-case” district-targeting scenario, and results from targeting strategies should be interpreted relative to one another.

Taking a logistically simpler approach to incidence-rate-based targeting, where high-risk districts within high-risk countries are targeted together (rate-logistics optimized), 25.3% (95% CI 24.8%–26.1%) of cases that would have otherwise occurred without vaccination were averted cumulatively, translating to 617,424 (95% CI 599,150–643,791) cases, 24,189 (95% CI 23,579–25,115) deaths, and 577,533 (95% CI 564,572–590,935) DALYs averted after 13 years of vaccination campaigns (Fig 2; Figs M–O and Table D in S1 Appendix).

Targeting districts geographically by lack of access to improved water and sanitation was not as effective as targeting by historical cholera disease burden. When targeting districts by lack of access to improved water (water optimized), 11.2% (95% CI 11.1%–11.4%) of cases were averted; this translates to 273,939 (95% CI 270,319–277,002) cases, 10,672 (95% CI 10,517–10,827) deaths, and 255,090 (95% CI 251,723–258,787) DALYs averted after 13 years of vaccination campaigns (Fig 2; Figs M–O and Table D in S1 Appendix). Targeting by lack of access to improved sanitation was substantially less effective (sanitation optimized); 4.5% (95% CI 4.3%–4.6%) of cases were averted, representing 109,817 (95% CI 103,735–114,110) cases, 3,682 (95% CI 3,469–3,812) deaths, and 83,228 (95% CI 78,579–86,117) DALYs that would have otherwise occurred without vaccination from 2018 through 2030 (Fig 2; Figs M–O and Table D in S1 Appendix).

Across all years with vaccination campaigns, the rate-optimized strategy averted 20%–47% of annual cholera cases (range of mean estimates across years from 2018 to 2030) and the rate-logistics-optimized strategies averted 9%–31% of annual cholera cases (Table E in S1 Appendix). Burden-based deployment strategies substantially outperformed the water-optimized and sanitation-optimized strategies, which averted 6%–14% and 2%–7% of annual cholera cases, respectively (Table E in S1 Appendix). The untargeted strategy, where vaccine was deployed at equal coverage across all districts, yielded a 0.7%–4% annual case reduction from 2018 to 2030 (Table E in S1 Appendix).

**Fig 3:**
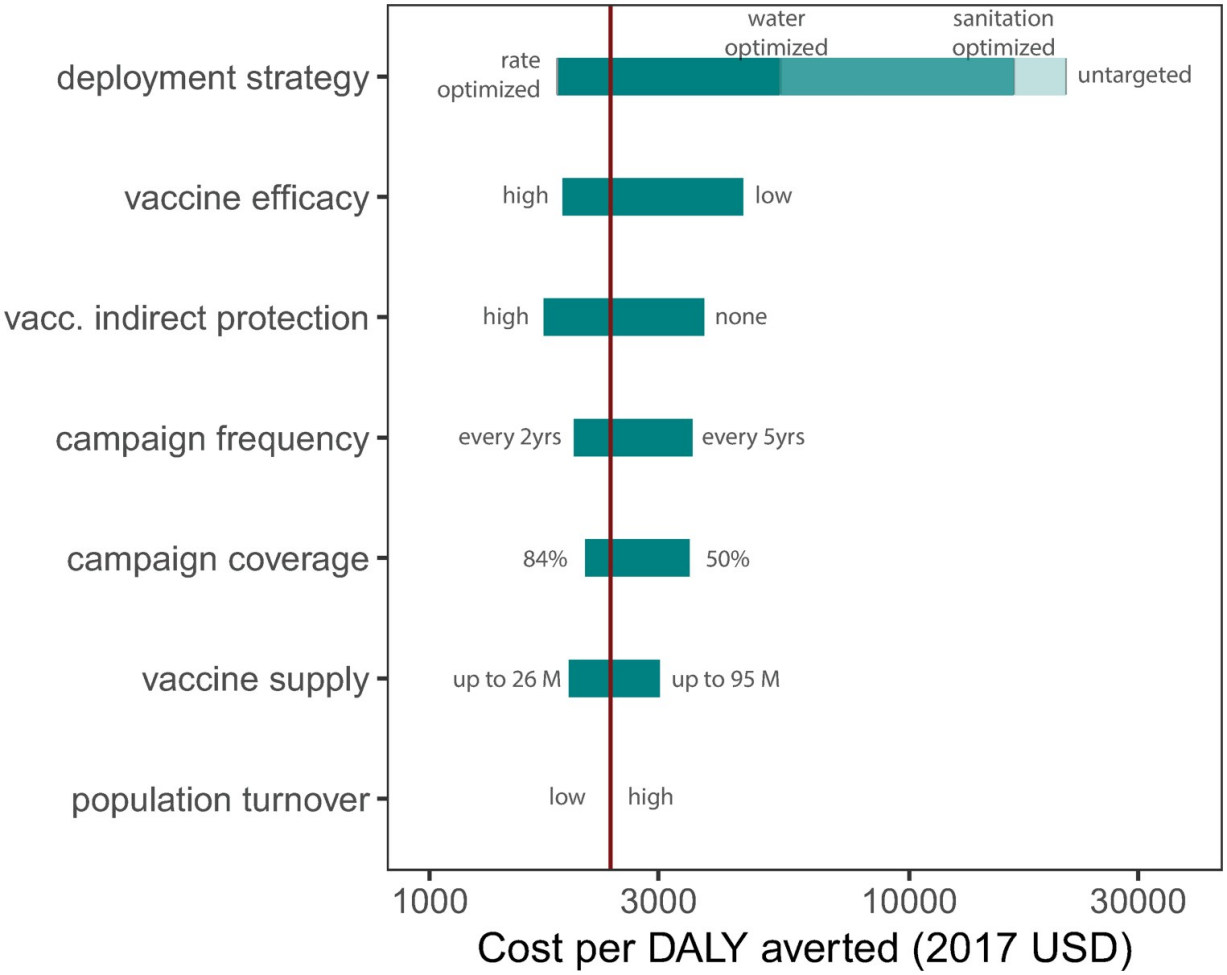
Analysis of sensitivity of the mean cost per DALY averted to alternate parameters for vaccination deployment strategy, vaccine efficacy, indirect vaccine protection, vaccination campaign frequency, vaccination campaign coverage, vaccine supply, and population turnover rate. The red vertical line indicates the mean cost per DALY averted for the rate-logistics-optimized scenario with the primary model parameters ($2,383). The untargeted and rate-optimized strategies represented the highest- and lowest-cost vaccination deployment strategies, respectively. DALY, disability-adjusted life year; USD, US dollars.

The most effective vaccination deployment strategies were also the most cost-effective in our simulations. We projected mean costs of $1,843 (95% CI 1,032–2,382) and $2,383 (95% CI 1,327–3,102) per DALY averted (2017 USD) for the rate-optimized and rate-logistics-optimized strategies, respectively (Fig 3; Table D in S1 Appendix). Targeting by risk factors was much more expensive; mean costs for the water-optimized and sanitation-optimized strategies were $5,394 (95% CI 3,029–6,965) and $16,546 (95% CI 9,121–22,243) per DALY averted (2017 USD), respectively (Table D in S1 Appendix). As a point of reference, the 2017 gross domestic products (GDPs) of countries within our study area ranged from roughly $300 to $10,000, with a mean around $1,734 (2017 USD); interventions are typically defined as cost-effective if the mean cost per DALY averted is less than 3 times the GDP of a country, and as highly cost-effective if it is less than or equal to the GDP of a country [34,37].

We examined the 1-way sensitivity of our results to alternate parameters for vaccine efficacy, indirect vaccine protection, vaccination campaign frequency, vaccination coverage, vaccine supply, population turnover, and campaign deployment strategy, taking the rate-logistics-optimized strategy as the primary scenario (Table 1; Fig 3). Changing the vaccination deployment strategy produced the greatest difference in mean cost per DALY averted: $1,843 (95% CI 1,032–2,382) versus $21,213 (95% CI 11,953–27,263) for the rate-optimized and untargeted strategies, respectively (Table D in S1 Appendix). Varying vaccine efficacy produced the second-largest difference among model parameters, where using the 97.5 and 2.5 percentile vaccine efficacy estimates (Table 1) from a recent meta-analysis resulted in mean costs per DALY averted of $1,892 (95% CI 1,053–2,463) and $4,507 (95% CI 2,512–5,864), respectively (Tables F and G in S1 Appendix). The model was nearly as sensitive to assumptions about indirect vaccine protection, where assumptions of high indirect protection and no indirect protection (Table 1) had mean costs per DALY averted of $1,729 (95% CI 963–2,250) and $3,733 (95% CI 2,079–4,859), respectively (Tables H and I in S1 Appendix). Model results were less sensitive to vaccination campaign frequency, vaccination coverage, vaccine supply, and population turnover rate; these results are reported in Tables J–Q in S1 Appendix.

## Discussion

This study suggests that geographic targeting is critical to ensuring that extended cholera vaccination campaigns across sub-Saharan Africa have a measurable impact on cholera incidence. When considering the direct and indirect protective effects of OCVs, our results suggest that, under projected resource constraints, campaigns that are geographically targeted according to disease burden may avert over 8 times more cholera cases, deaths, and DALYs than untargeted (i.e., general population) approaches. Vaccination deployment strategy can have a greater impact on health impact and cost-effectiveness than substantial improvements to vaccine efficacy, vaccination campaign frequency, and vaccination coverage.

Recent discussions and guidance by the GTFCC have suggested that priority areas for preventive cholera control (burden hotspots) should be ranked according to mean annual incidence, and that risk factors like access to water and sanitation should have a secondary role. Decision-making around OCV allocation and targeting is complex, and different actors are concerned with maximizing public health impact, navigating global and local politics, and considering the ethics of balancing preventive, responsive, and humanitarian uses of vaccine. Our results contribute to the discussion around maximizing the public health impact for preventive and potentially routine OCV campaigns, but our results should not be generalized to identify locations at risk for new cholera introductions. Seventy percent of the targets in the best-performing strategy are in countries with endemic cholera, and it is likely that reactive campaigns would occur in locations with less predictable cholera patterns (Table C in S1 Appendix). While it is unlikely that OCV targeting decisions will occur on a continental scale in the real world, we sought to examine the epidemiologic principles that will likely be considered in future OCV policy discussions.

Two vaccination deployment strategies with different disease-burden-based criteria for identifying high-risk districts in Africa yielded similarly effective results. We believe the rate-logistics-optimized approach to be the most practical deployment strategy considered. In this approach, countries are ranked and selected by population living in high-risk districts, and then only the high-risk districts within selected countries are targeted for vaccination. While the less practical rate-optimized strategy (which prioritizes districts based on burden regardless of country) does prevent more cases, the resulting benefit would not likely outweigh the unmeasured added costs in logistical implementation. Our study suggests that vaccination targeting the highest-risk geographic locations can be a cost-effective cholera treatment strategy, thus complementing more comprehensive analyses that suggest the cost-effectiveness of targeting high-risk demographic groups [38].

Improved access to safe water and sanitation is necessary for long-term cholera control and reductions in overall diarrheal disease burden [39–41]. We examined the impact of vaccination deployment strategies that targeted districts with the lowest access to improved water and sanitation as measured by JMP indicators [14,15], since districts with the poorest access to improved water and sanitation are not the same as those with the highest historical cholera burden. Targeting by water and sanitation access for the same number of deployed vaccines averted less than one-third as many cholera cases as targeting by disease-burden-based criteria (Table D in S1 Appendix). However, our analyses were based on indicators that have been criticized for their focus on access to water and sanitation infrastructure as opposed to safely managed and sustainable water and sanitation use [42], which may explain the relatively poor performance of the water- and sanitation-optimized strategies. The JMP recently adopted new measurement criteria, which may prove to be more specific indicators of cholera risk [43].

We examined the sensitivity of our model to different assumptions about cholera and cholera vaccine dynamics in our models, but there remain several limitations outside the scope of these analyses (Tables F–Q in S1 Appendix). Our models do not account for immunity due to natural cholera infection, and the indirect effects of vaccination are captured only at the grid-cell level, which limits the estimated impact of vaccination at critical hubs of cholera transmission. Our projections assume that baseline cholera incidence (measured as mean annual incidence) remains constant throughout the study period, which may not capture secular trends, inter-year variability in cholera incidence or surveillance, or the emergence of new cholera patterns due to conflict, crisis, or changing epidemiology. The uncertainty in our model results reflects only variability in baseline cholera risk (not fully specified parameter uncertainty) and therefore remains underestimated, but mean annual incidence appears to be an unbiased estimator for annual incidence (Fig K in S1 Appendix), suggesting that our results are valid in the expectation. Additionally, our baseline incidence estimates represent only reports of suspected cholera cases, making no explicit adjustments for biased reporting or measurement error. Further, we examined only the impact of 2-dose vaccination campaigns; recent evidence suggests that a single OCV dose may be efficacious [8,44–46], but few studies have presented data on long-term protection relative to a 2-dose course. While the inclusion of age-specific parameters in our model might be biologically justified, there is a dearth of age-specific cholera incidence data and long-term vaccine efficacy estimates in children [8].

Improvements to the coverage and geographic resolution of cholera surveillance, and an improved understanding of the relationship between cholera, climate, and disruptive events, may enable future versions of this model to characterize optimal OCV targeting for epidemic and endemic settings. Future water, sanitation, and hygiene (WASH) survey data collected under the new JMP definitions, combined with higher quality cholera surveillance data, may make nuanced treatment of complex epidemiologic interactions possible. For instance, projections of OCV impact could employ more complex targeting strategies that account for interactions between cholera immunity due to recent outbreaks and the probability of epidemic cholera introduction and propagation due to limited WASH access.

Our results show how geographic targeting can play an essential role in ensuring that mass OCV use leads to substantial reductions in the global burden of cholera, even under current supply constraints. Strategic targeting of resources can play an essential role in making cholera control efforts cost-effective, a message that may be generalized to the entire suite of cholera control activities, including those to increase access to safe water and improved sanitation. Continued increases in global OCV production would enable a greater proportion of high-risk populations to be targeted with vaccination with greater regularity, but our results suggest that even the most effective geographic targeting strategies paired with optimistic OCV supply projections will not be enough to achieve the global cholera burden reduction goals set forth by the World Health Assembly resolution and the GTFCC roadmap to 2030. Substantial improvements to other sectors of the roadmap, including broad investments and progress in improving water and sanitation infrastructure and cholera surveillance, will be needed to make headway in this ambitious initiative.

## Supporting information

S1 Appendix Detailed information on the model assumptions, the calculation of public health impact and costs, sensitivity analyses, and supporting analyses. This content includes Tables A to Q and Figs A to O.
(PDF)

## Abbreviations

DALY: disability-adjusted life year
GTFCC: Global Task Force on Cholera Control
JMP: WHO/UNICEF Joint Monitoring Programme for Water Supply, Sanitation and Hygiene
OCV: oral cholera vaccine
USD: US dollars
watsan: water and sanitation
