# Supplementary material for: The projected impact of geographic targeting of oral cholera vaccination in sub-Saharan Africa: A modeling study
Source: PLoS Med. 2019 Dec 11;16(12):e1003003. doi: 10.1371/journal.pmed.1003003 (PMC6905526; doi:10.1371/journal.pmed.1003003)
Supplement: S1 Appendix — This content includes Tables A to Q and Figs A to O. (PDF) [file pmed.1003003.s001.pdf]

The projected impact of geographic targeting of oral cholera vaccination in sub-Saharan Africa: a modeling study

Supplementary Material

Elizabeth C. Lee, Andrew S. Azman, Joshua Kaminsky, Sean M. Moore, Heather S. McKay, Justin Lessler

Contents

Vaccination Campaign Coverage 2
Vaccine Efficacy 3
Vaccine Indirect Effects 4
Vaccine Supply Projections 5
Vaccination Deployment Strategies 6
Measuring Public Health Impact and Cost Benefit 9
Vaccination Campaign Costs 11
Constant Incidence Assumption 12
Percentage of Targets in Epidemic Countries 13
Baseline Model Outcomes 14
Summary Tables by Parameter Set 16
    Baseline 16
    Vaccine Efficacy 18
    Indirect Vaccine Protection 20
    Campaign Frequency 22
    Campaign Coverage 24
    Vaccine Supply 26
    Population Turnover 28

Vaccination Campaign Coverage

We assessed the sensitivity of our models to vaccination campaign coverage, as there has been great variability in the success of previous OCV campaigns. We first conducted a review of published literature on post-OCV campaign vaccination coverage surveys and identified seven studies related to 24 two-dose campaigns conducted globally from 2003 through 2016 (Table A) (Lam et al. 2017; Tohme et al. 2015; Luquero et al. 2013; Uddin et al. 2014; Massing et al. 2018; Sema Baltazar et al. 2018; Cavailler et al. 2006).

Table A: **Published coverage survey estimates from previous OCV campaigns.** Median point estimates and standard errors were used to resample coverage from a normal distribution for each campaign.

Study	Campaign	Coverage	SE	Sample Size
Lam 2017	Dahuk 2-dose, Iraq	0.900	0.0255102	390
Lam 2017	Erbil 2-dose, Iraq	0.930	0.0153061	512
Lam 2017	Sulaymaniya 2-dose, Iraq	0.930	0.0178571	552
Lam 2017	Anbar 2-dose, Iraq	0.980	0.0153061	550
Lam 2017	Wasit 2-dose, Iraq	0.910	0.0255102	575
Lam 2017	Salah Addin 2-dose, Iraq	0.810	0.0306122	652
Lam 2017	Najaf 2-dose, Iraq	0.740	0.0357143	773
Lam 2017	Baghdad Karkh 2-dose, Iraq	0.370	0.0382653	1273
Lam 2017	Kerbala 2-dose, Iraq	0.300	0.0408163	1763
Lam 2017	Babil 2-dose, Iraq	0.210	0.0306122	2405
Tohme 2015	Petit Anse 2-dose, Haiti	0.625	0.0229592	1118
Tohme 2015	Cerca Carvajal 2-dose, Haiti	0.768	0.0272959	809
Luquero 2013	Boffa 2-dose, Guinea	0.780	0.0459184	840
Luquero 2013	Douprou 2-dose, Guinea	0.760	0.0408163	541
Luquero 2013	Koba 2-dose, Guinea	0.690	0.0459184	935
Luquero 2013	Mankountan 2-dose, Guinea	0.840	0.0357143	576
Luquero 2013	Tamita 2-dose, Guinea	0.780	0.0510204	205
Luquero 2013	Tougnifili 2-dose, Guinea	0.770	0.0561224	826
Luquero 2013	Kaback 2-dose, Guinea	0.740	0.0357143	764
Luquero 2013	Kakossa 2-dose, Guinea	0.780	0.0459184	113
Uddin 2014	Dhaka second dose, Bangladesh	0.790	0.0267857	39910
Massing 2018	Kalemie Strata 1 2-dose, DR Congo	0.672	0.0257653	1159
Sema Baltazar 2018	Nampula, card and oral report, 2-dose, Mozambique	0.512	0.0673469	648
Cavailler 2006	Beira, 2-dose, Mozambique	0.536	0.0153061	82381

The study site coverage estimates were skewed slightly left due to the presence of outlying low vaccination campaigns among our study pool (with a mean of 71.3% and median of 76.9%).

For each of the seven studies, we resampled two-dose (the standard vaccine regimen) coverage estimates 5000 times from a Gaussian distribution with a mean equal to the estimated coverage at the campaign site and the variance derived from the associated 95% confidence intervals; for studies with multiple locations, we first drew a single location randomly and then sampled from a Gaussian distribution of coverage estimates for that location. We pooled these 35,000 draws across studies and used the median (68%) of the samples as the baseline coverage estimate for our model (Figure A). The 10th (49%) and 90th (84%) percentiles of the resampled distribution were used for the low and high coverage sensitivity analyses. These estimates give studies equal weight to the distribution regardless of the number of campaign locations.

As there are many possible methods for identifying OCV coverage parameters for our models, we compared the parameters we used in our study (shown above), to others that could have been proposed. As one alternative, we performed the resampling exercise where weights were applied according to campaign site sample size (Figure A). The median was 66%, and the 10th and 90th percentiles were 44% and 82% respectively. As a second alternative, we performed the equal-weighting sampling exercise again under the assumption that

coverage should follow a lognormal distribution (Figure A). The median was 68%, and the 10th and 90th percentiles were 49% and 84% respectively.

The percentiles of interest were the same up to the 100th decimal place for the unweighted Gaussian (used in the main text) and lognormal resampling approaches, while the weighted Gaussian resampled percentiles were slightly lower than those from the unweighted Gaussian resampling scheme.

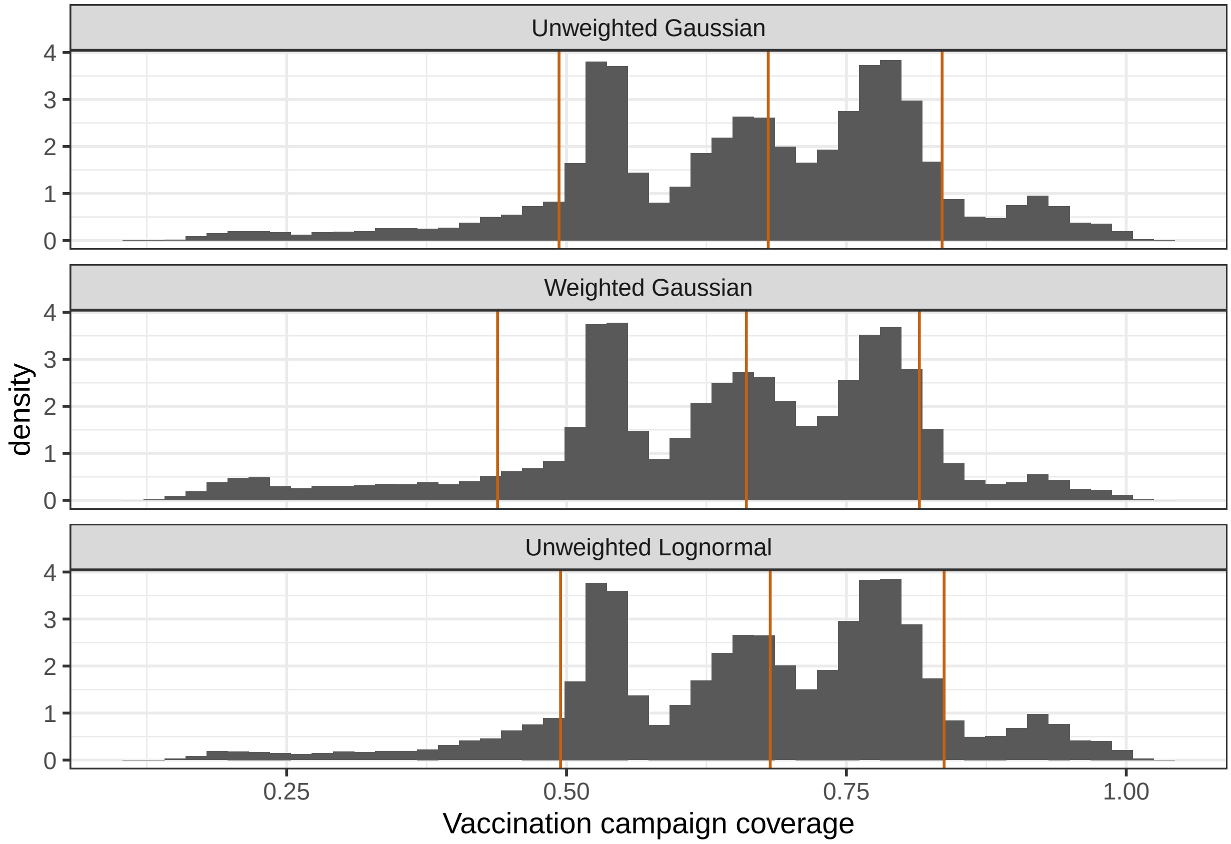

Figure A: **Resampled distributions of OCV campaign coverage, with Gaussian assumptions and equal weights across campaign sites, which was used for the main paper (top), with Gaussian assumptions and weights for campaign site sample size (middle) and with lognormal assumptions and equal weights across campaign sites (bottom).** We generated a distribution of OCV campaign coverage estimates for each resampling scheme from published OCV coverage surveys. Rust orange lines indicate the 10th percentile, median, and 90th percentile of the resampled distributions.

Vaccine Efficacy

We examined the sensitivity of our OCV health impact estimates to different vaccine efficacy assumptions. We fit a log-linear decay function to two-dose vaccine efficacy data reported zero to five years after vaccination in a recent meta-analysis (Bi et al. 2017); study estimates were weighted by the inverse of the squared standard error in the model. We then used the mean point estimates for each year as (direct) vaccine efficacy in our model. In this framework, the initial vaccine efficacy is 66% declining to 0% after six years (Figure B). We modeled vaccination with only the full two-dose regimen with no wastage.

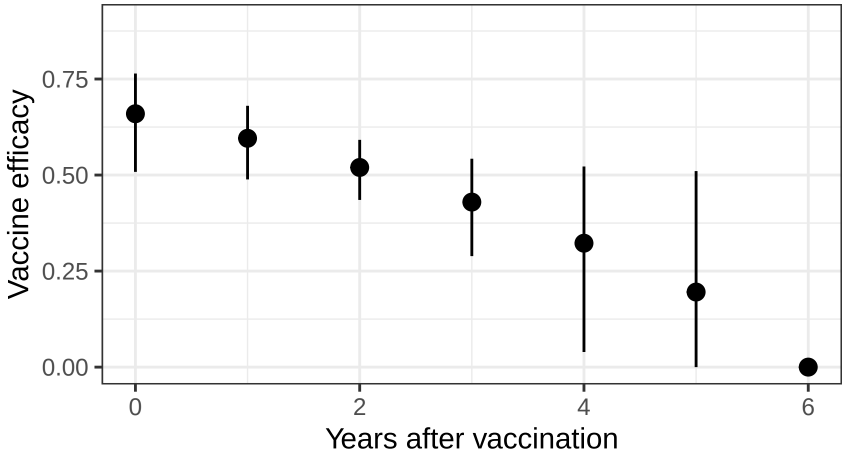

Figure B: **Waning vaccine efficacy in years after vaccination.** The data displayed represents the mean point estimate and 95% confidence intervals across data collected across seven vaccine efficacy studies. Mean point estimates were used to parametrize the primary model, while the 2.5 and 97.5 percentiles were used to parametrize the low and high vaccine efficacy models, respectively.

Vaccine Indirect Effects

Due to limited data and uncertainty about the spatial scale of indirect vaccine protection, we assessed the sensitivity of our modeled health impacts to different indirect vaccine protection assumptions. For our baseline parameter set, we modeled indirect protection as a function of the vaccination coverage in a given grid cell using data from trials in India and Bangladesh (Ali et al. 2005, 2013). Specifically, the phenomenological association between the relative reduction in incidence among unvaccinated (placebo) individuals and OCV coverage in their "neighborhood" was fit to a logistic function (Figure C). Under this model of indirect vaccine protection, individuals not protected by vaccine and residing in grid cells with 50% and 70% vaccination coverage experienced an 80% and near 100% reduction in cholera risk compared to no vaccination scenarios, respectively.

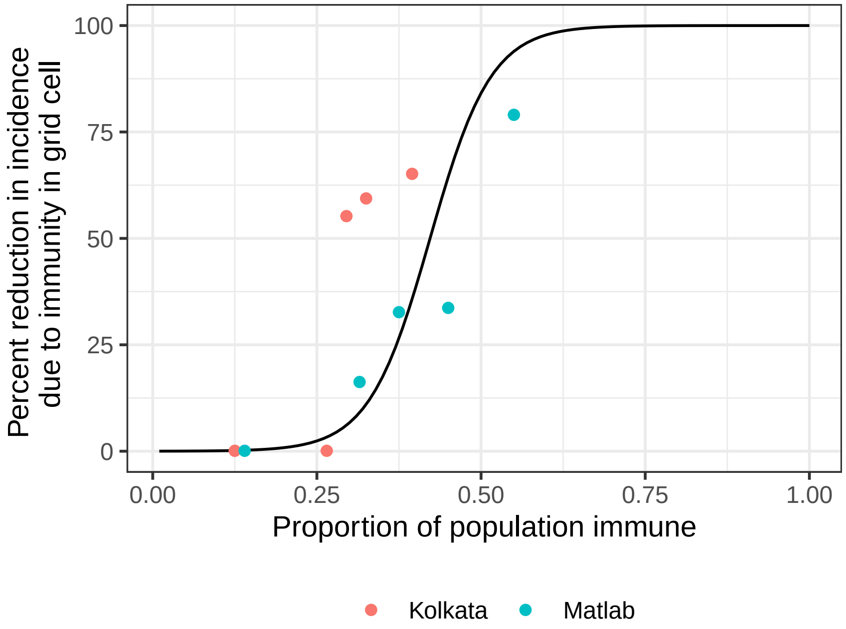

Figure C: **Indirect protective effect of vaccination.** Each point represents the percent reduction in incidence due to cholera vaccination coverage (population immunity), as reported by randomized controlled trials in Kolkata and Matlab. The black line represents the logistic function fit to these data.

Sensitivity parameters for indirect vaccine effects assumed, at the lower end, that vaccination conferred no indirect protection. On the upper end, we assumed that individuals not protected by vaccine and residing in grid cells with 30%, 50%, and 70% vaccination coverage experienced a respective 66%, 88%, and 97% reduction in cholera risk, according to a logistic model fit of published estimates (Figure D) (Longini Jr. et al. 2007).

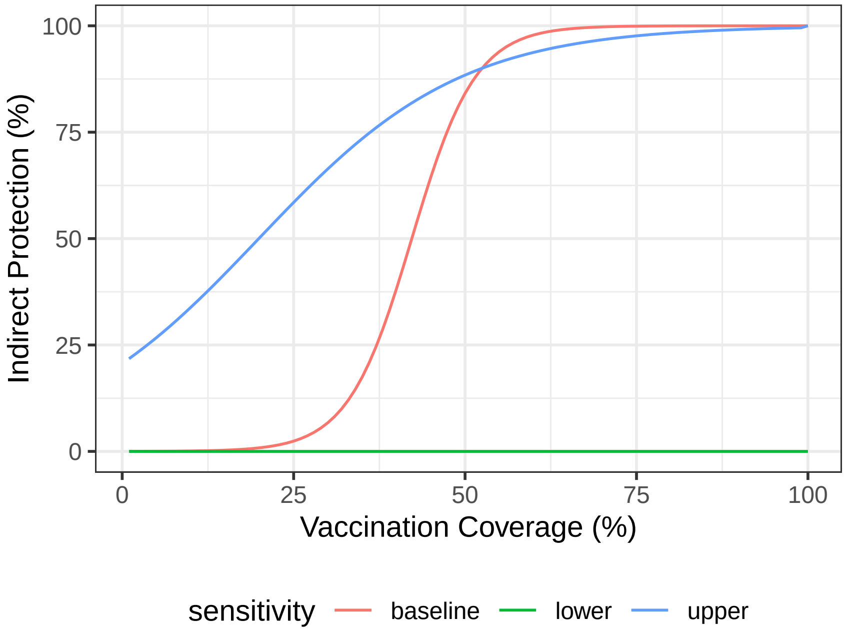

Figure D: **Sensitivity parameters for indirect protective effect of vaccination.**

Vaccine Supply Projections

There is great uncertainty in OCV supply over the next decade; increasing demand has led to plans to open several new manufacturing facilities, but the timing of these projected increases in supply remains uncertain. Based on estimates from experts within the GTFCC and data from vaccine manufacturers in the first half of 2018, we assumed that global OCV supply would increase linearly from 23 million doses in 2018 to 59 million doses in 2030 (Figure E).

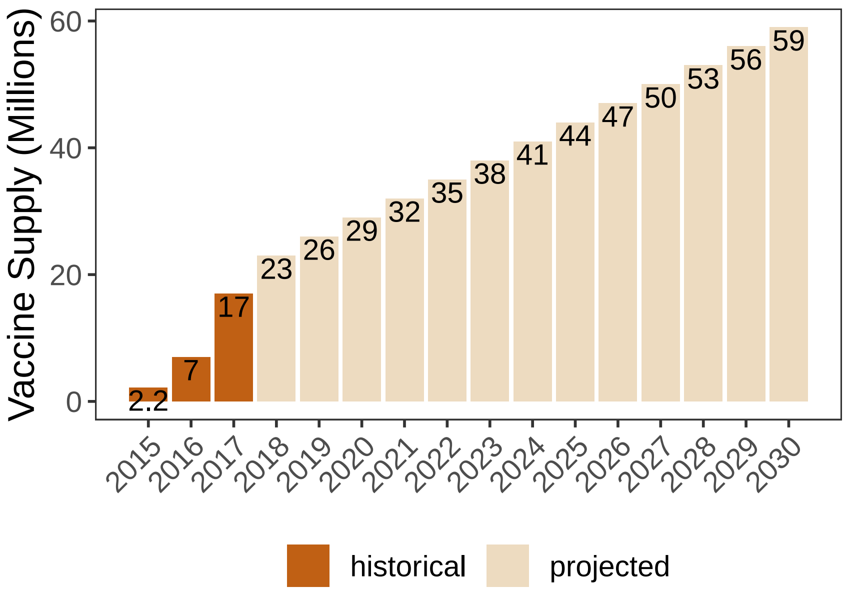

Figure E: **The historical (2015-2017) and projected (2018-2030) cholera vaccine supply.** The projected vaccine supply per year (in millions) was used as a model input.

Sensitivity parameters for vaccine supply assumed after 2019 either no growth or linear growth to 95 million doses in 2030; upper limit vaccine supplies may be achieved if new OCV production facilities open as planned (Figure F).

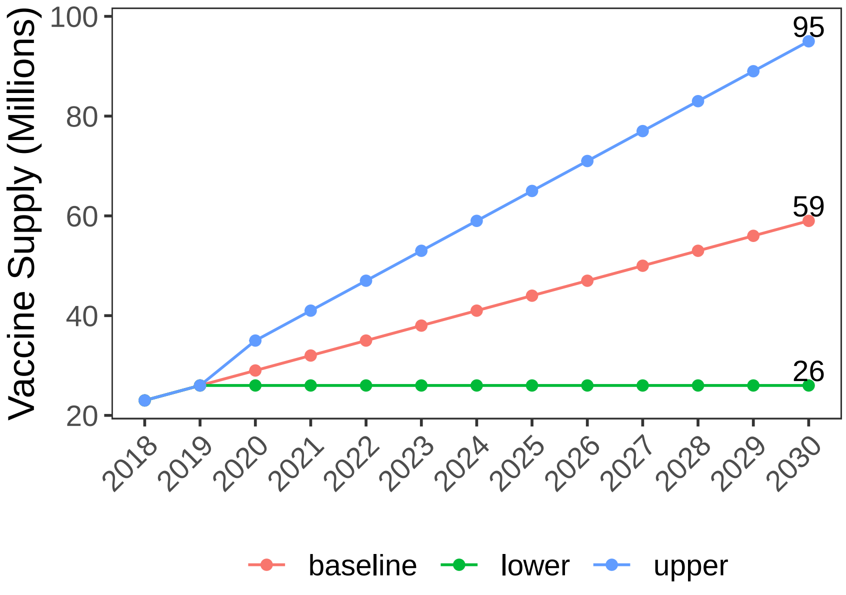

Figure F: **Sensitivity parameters for projected cholera vaccine supply (in millions) from 2018-2030.**

Vaccination Deployment Strategies

The case optimized, case-logistics optimized, and watsan optimized results were not reported in the main text. The case optimized strategy performed worse than the rate optimized but better than the rate-logistics

optimized strategies; 28.7% (95% CI: 26.6-29.7%) of cases that would have otherwise occurred without vaccination from 2018 through 2030 were averted. This reduction translates to 698,000 cases, 28,000 deaths, and 657,000 DALYs averted after 13 years of vaccination. In the case-logistics optimized strategy, 25.4% (95% CI: 24.5-26.4%) of cumulative cases that would have otherwise occurred without vaccination were averted, thus translating to 619,000 cases, 24,000 deaths, and 581,000 DALYs averted after 13 years of vaccination.

The watsan optimized strategy was similar to the water optimized and sanitation optimized strategies, but the districts were prioritized according to those with the lowest access to improved water or sanitation. As a combination of the water and sanitation optimized strategies, the watsan optimized strategy performed better than the sanitation optimized strategy and less well than the water optimized strategy; 8.2% (95% CI: 7.7-8.4%) of cases that would have otherwise occurred without vaccination from 2018 through 2030 were averted, which translates to 199,000 cases, 7,000 deaths, and 171,000 DALYs averted after 13 years of vaccination campaigns.

The rate optimized and rate-logistics optimized vaccination campaign deployment strategies are described in greater detail in Figure G and Figure H.

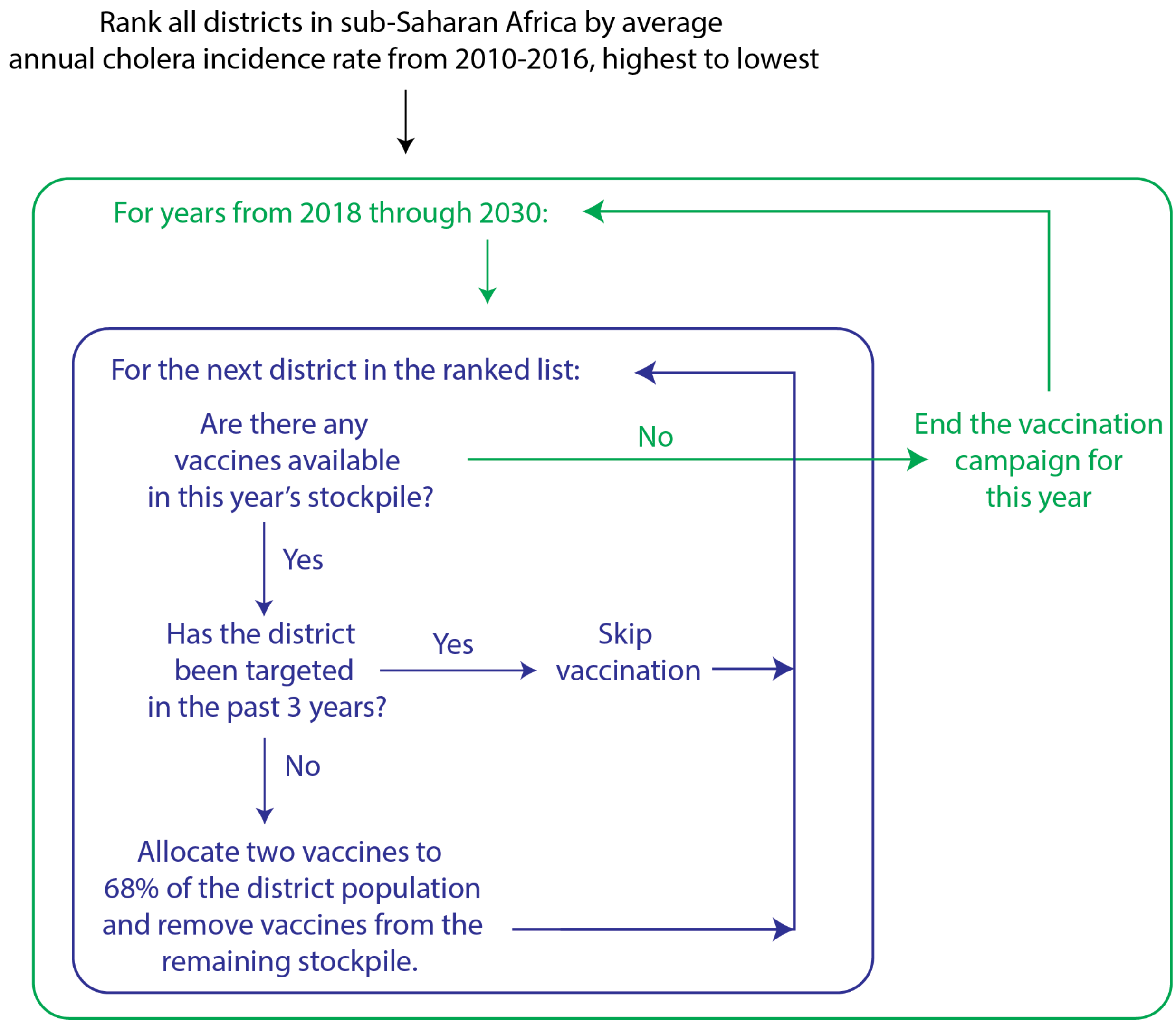

Figure G: **Flowchart depicting the rate optimized vaccination deployment strategy with primary scenario parameters.**

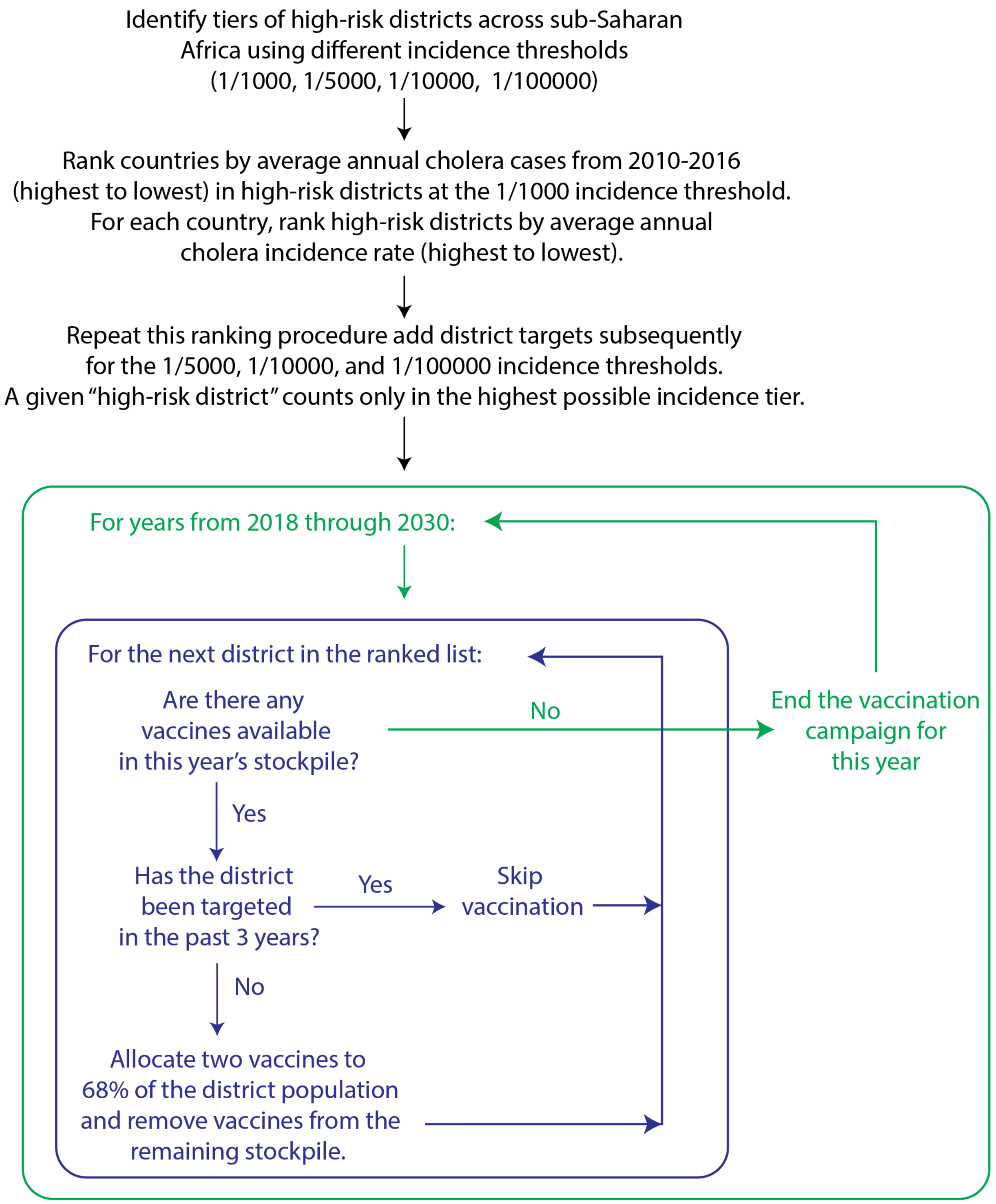

Figure H: **Flowchart depicting the rate-logistics optimized vaccination deployment strategy with primary scenario parameters.**

Measuring Public Health Impact and Cost Benefit

We calculated the number of cases averted as the difference in cases between vaccination and no vaccination scenarios. To estimate the number of deaths averted, we multiplied a country-specific case-fatality ratio (CFR) with the expected cases outputs. We estimated country-level CFRs as the ratio of the total number of deaths to cases across all years where cholera cases were reported in the WHO Global Health Observatory database from 1970 to 2016 among countries in our study region (Figure I). For countries where the estimated CFR exceeded 7% (often based on outbreaks with a small total number of cases), we applied the mean CFR across all remaining countries (3.4%).

We also estimated the disability-adjusted life-years (DALYs) for the vaccination and no-vaccination scenarios, where DALYs are defined as the sum of years of life lost (YLLs) and years of life disabled (YLDs). YLLs were calculated as

$$YLL_i = CFR_i(Y_{i,t} - Y*_{i,t})(\kappa_{i,t-\rho_i} - \rho_i),$$

where ρ_i is the average age of cholera infection in location i and $\kappa_{i,t-\rho_i}$ is the average life expectancy for someone of average cholera infection age in year t. Country-level estimates of life expectancy for each projected year in our study period were obtained from the United Nations (United Nations Secretariat, Department of Economic and Social Affairs, Population Division 2017) and average age of cholera infection was calculated as the inverse-variance-weighted mean age among cases identified in OCV trials in Africa and the Caribbean (25.75 years old) (Azman et al. 2016; Ferreras et al. 2018; Luquero et al. 2014; Khatib et al. 2012). YLDs were calculated as the product of the proportion of the year disabled with illness (assumed at 4/365) and a disability weight for severe diarrheal disease as estimated from the 2016 IHME Global Burden of Disease Study (0.247) (Institute for Health Metrics and Evaluation, Global Burden of Disease Collaborative Network 2017). Disability weights range from 0 to 1 and represent the magnitude of the health loss associated with a specific health status.

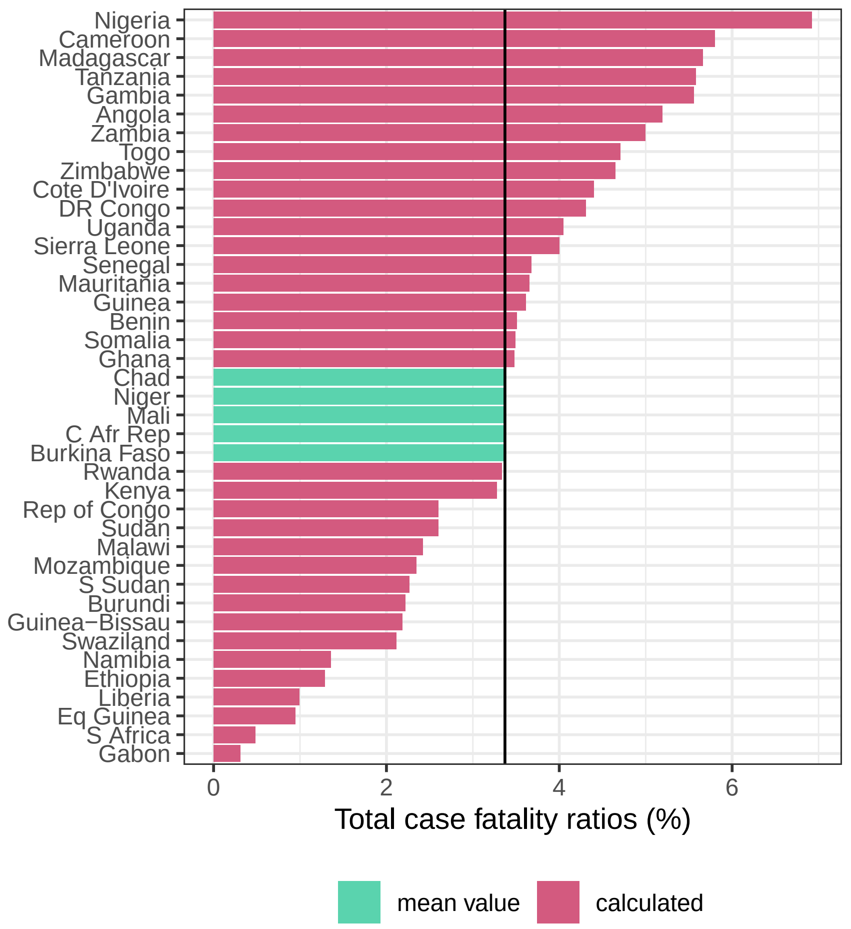

Figure I: **Case fatality ratios pooled across all available data by country.** We calculated the total case fatality ratios by country for all years of data available in the WHO Global Health Observatory database. For a given country, data could have been reported for any year within the range of 1970 through 2016. For countries with implied case fatality ratios greater than 7% (colored in seafoam green), we used the mean of the case fatality ratios below 7% (3.4%).

We compared the country-level total (pooled across years) CFR estimates to the outputs from country-specific random effects models, where CFR observations were computed annually with all available WHO Global Health Observatory data (Figure J). We found that the estimates from the random effects models were systematically higher than the total CFRs. We preferred to use the total CFR estimates because they would generate more conservative estimates in the number of deaths averted through vaccination campaigns.

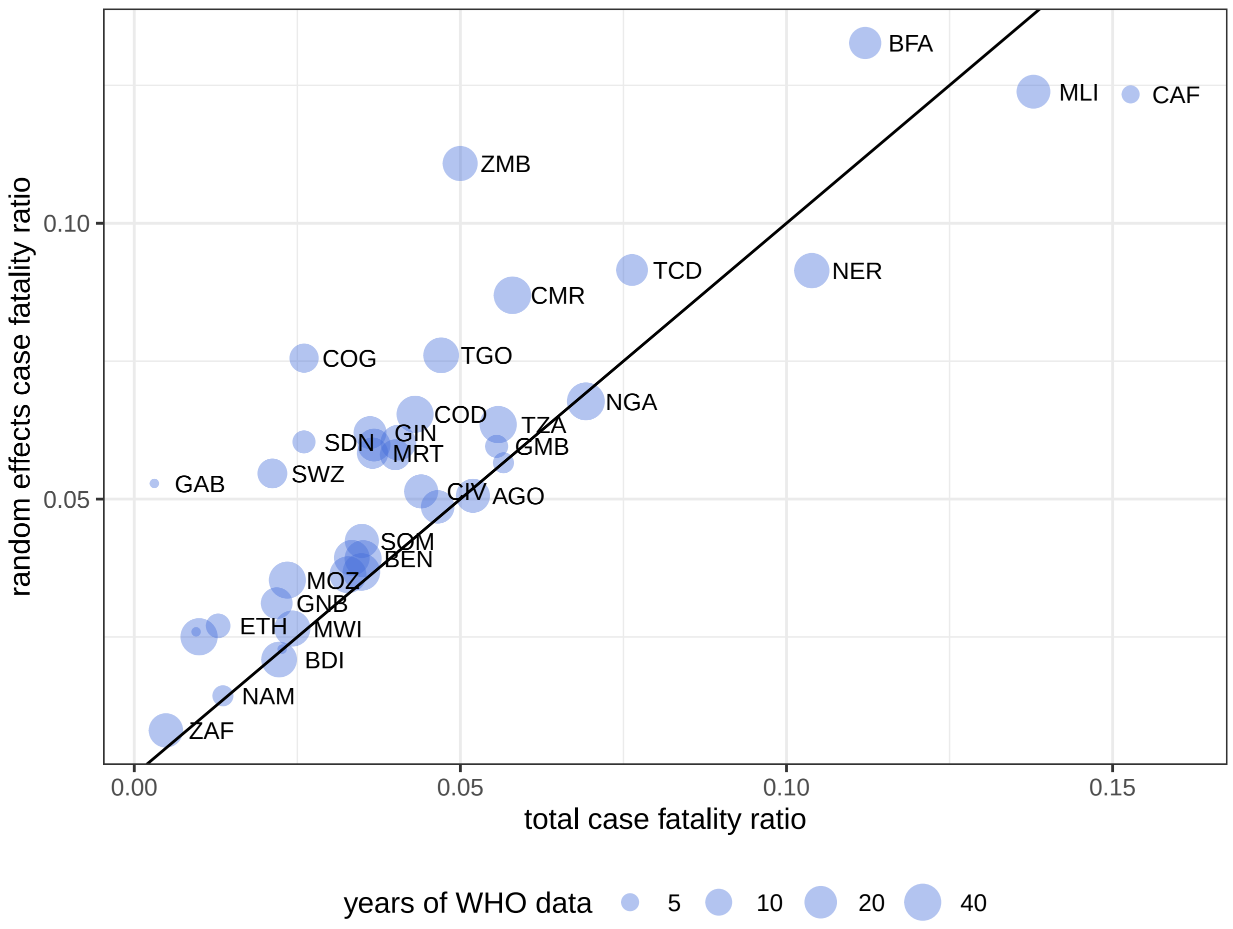

Figure J: **Comparison of total CFR and random effects model CFR estimates by country.** Random effects model estimates were systematically larger than total CFR estimates, and they were specifically higher in several countries where rate-logistics optimized targeting takes place, such as DR Congo (COD), Cameroon (CMR), and Sierra Leone (SLE).

Vaccination Campaign Costs

We reviewed four cost surveys for mass OCV campaigns that reported the vaccine delivery and vaccine procurement costs per fully vaccinated person (i.e., receiving two vaccine doses) in non-refugee African settings (Ilboudo and Le Gargasson 2017; Ciglenecki et al. 2013; Cavailler et al. 2006; Schaetti et al. 2012) (Table B). We excluded the estimate from Schaetti et al. 2012, due to its excessively high vaccine purchase prices. We felt that the exclusion of this study was reasonable based on current policy discussions, which suggest that future, preventive use of OCV in these settings are likely to be made available lower, negotiated rates.

All costs were adjusted to 2017 US dollars (USD) according to the World Bank Consumer Price Index. We used the mean of the three remaining cost survey data points ($6.32 per FVP), to calculate the cost per DALY averted results.

Table B: **Cost survey estimates (adjusted to 2017 USD) per fully vaccinated person (FVP) for previous vaccination campaigns in Africa.**

Study	Country	Procurement	Delivery	Total
Ilboudo and Le Gargasson 2017	Malawi	5.32	1.98	7.30

Study	Country	Procurement	Delivery	Total
Ciglenecki et al. 2013	Guinea	5.66	2.41	8.07
Cavailler et al. 2006	Mozambique	1.33	2.26	3.59

Constant Incidence Assumption

Our model assumed that baseline cholera incidence risk remained constant in our projections and we examined the reasonableness of this assumption using data from country-level annual cholera reports to WHO. First, we gathered all available country-level cholera case data from WHO annual cholera reports, which are reported annually in issues of the Weekly Epidemiological Record, for African countries in our study. Cholera case data were available from 1970 through 2017, but data are not complete for all countries and years.

We also gathered country-level population estimates from the 2019 United Nations World Population Projections, which were available in five-year intervals from 1950 through 2020. We fit a log-linear model to data for each country in order to obtain annual country-level estimates, and then we divided the case and population data to obtain annual estimates of the cholera incidence rate by country and year.

For each country-year pair from 2008-2017, we then compared the observed incidence rate to the expected incidence rate, which was calculated as the country's mean annual incidence rate across all remaining years in 10-year range (Figure K). The mean of the difference between observed and expected incidence rates (i.e., bias), was nearly zero (1.5E-16); this means that the mean annual incidence was an unbiased predictor of incidence in the held-out year, suggesting that our analysis is valid in the expectation. In addition, there was a positive relationship between the expected and observed incidence rates ($H_0 : \rho = 0$; $\rho = 0.31$, p-value $= 2.4E - 6$).

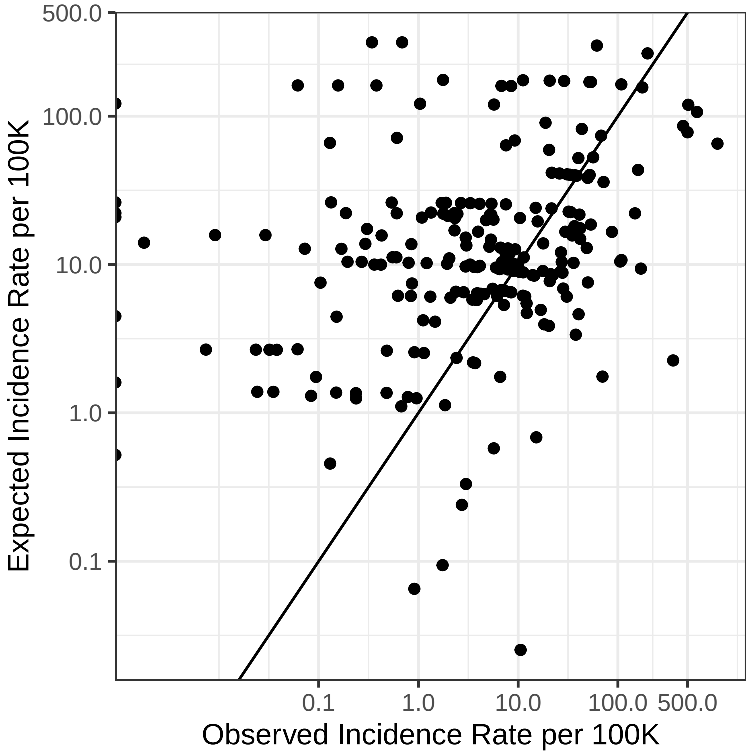

Figure K: **Observed versus expected mean annual incidence for country-year pairs according to WHO Annual Cholera Reports, 2008-2017.**

Percentage of Targets in Epidemic Countries

We examined the percentage of targets in the rate optimized, rate-logistics optimized, water optimized, and sanitation optimized strategies that occurred in countries with a coefficient of variation greater than 1.5 in their annual incidence rate from 2008 to 2017. We expect that countries with large coefficients of variation experience "epidemic" (instead of "endemic") cholera epidemiology, according to a framework previously proposed in Figure 4 of Lessler and Moore et al. (2018).

Table C: **Percentage of targets in epidemic countries for baseline parameters by vaccination deployment strategy.** Epidemic countries are defined as those with a coefficient of variation in annual incidence greater than 1.5 from 2008-2017, according to cholera data from the WHO annual cholera reports and population data from the UN World Population Prospects.

Deployment Strategy	Total Targets	# Epidemic Targets	% Epidemic Targets
case-logistics optimized	225	65	28.9
rate-logistics optimized	217	59	27.2
case optimized	236	69	29.2
rate optimized	227	67	29.5
sanitation optimized	253	40	15.8
watsan optimized	273	4	1.5
water optimized	333	19	5.7

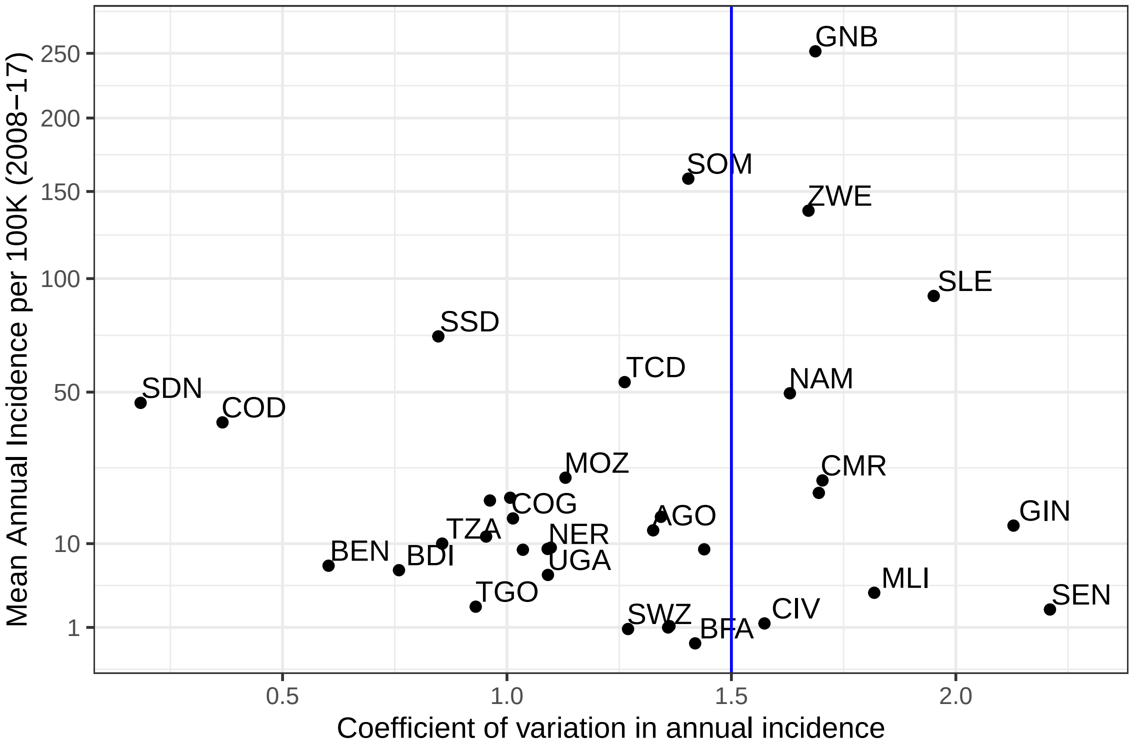

Figure L: **Cholera incidence versus the coefficient of variation of the annual incidence.** Mean annual incidence per 100,000 people versus the coefficient of variation of the annual incidence from 2008 to 2017 for 35 African countries based on reports to WHO. Countries with one or fewer years of reported data in this period were excluded. The blue line represents the threshold used to delineate endemic (left) and epidemic (right) countries.

In both the rate optimized and rate-logistics optimized strategies, 26% of locations receiving vaccine were located in countries with epidemic dynamics, respectively. In the water optimized and sanitation optimized

strategies, 6% and 16% of locations receiving vaccine were located in countries with epidemic dynamics, respectively. These analyses appear in the Rmd supplement file under Percentage of Targets in Epidemic Countries.

Baseline Model Outcomes

This section presents cumulative cases averted, deaths averted, and DALYs averted for the baseline parameter models for all vaccination deployment scenarios (Figure M, Figure N, Figure O).

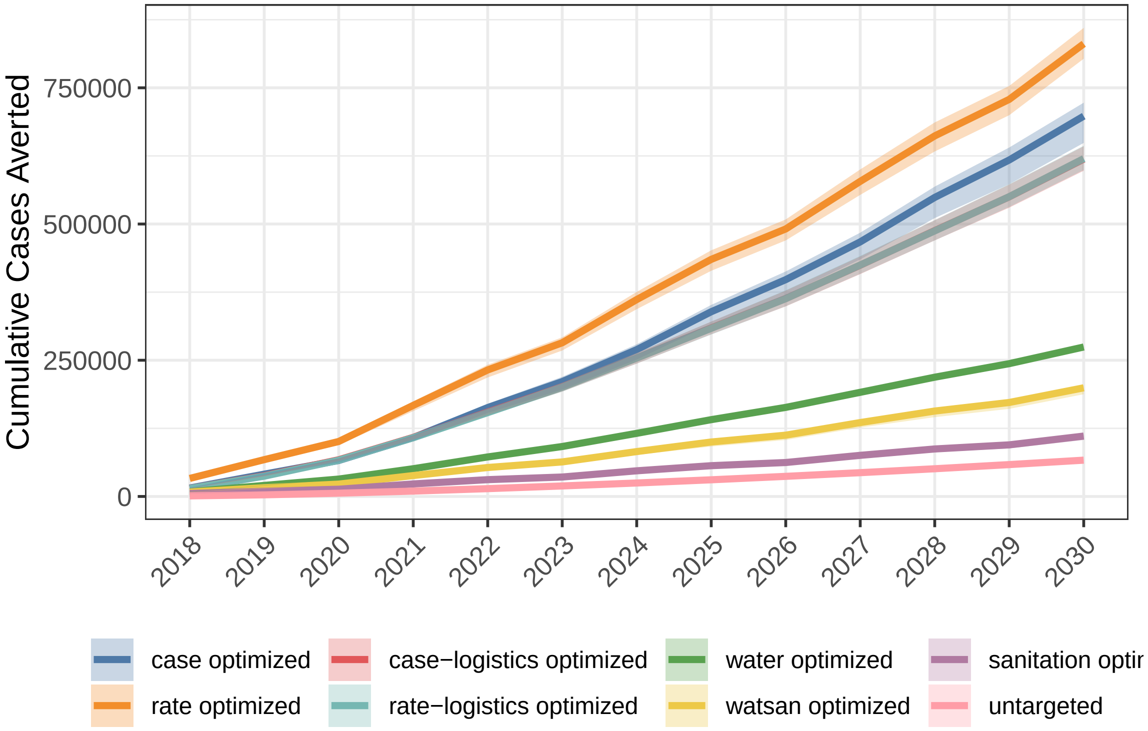

Figure M: **Cumulative cases averted from mass oral cholera vaccination campaigns across all vaccination deployment strategies in sub-Saharan Africa from 2018 through 2030.**

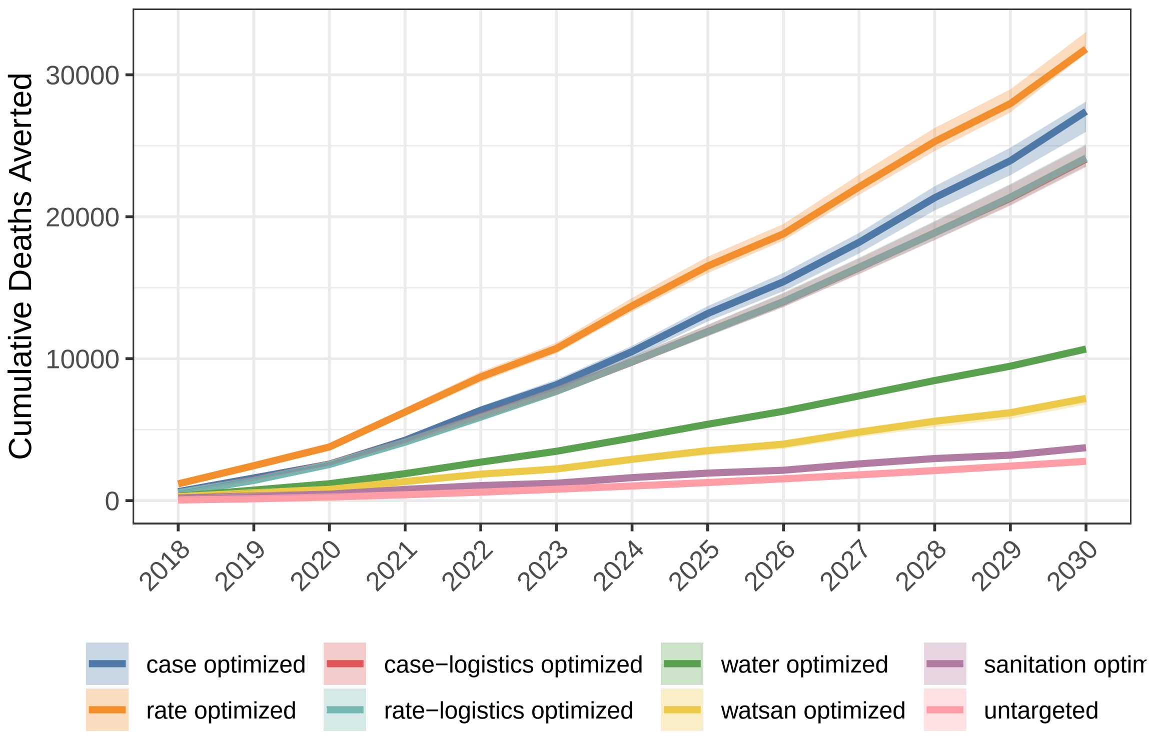

Figure N: **Cumulative deaths averted from mass oral cholera vaccination campaigns across all vaccination deployment strategies in sub-Saharan Africa from 2018 through 2030.**

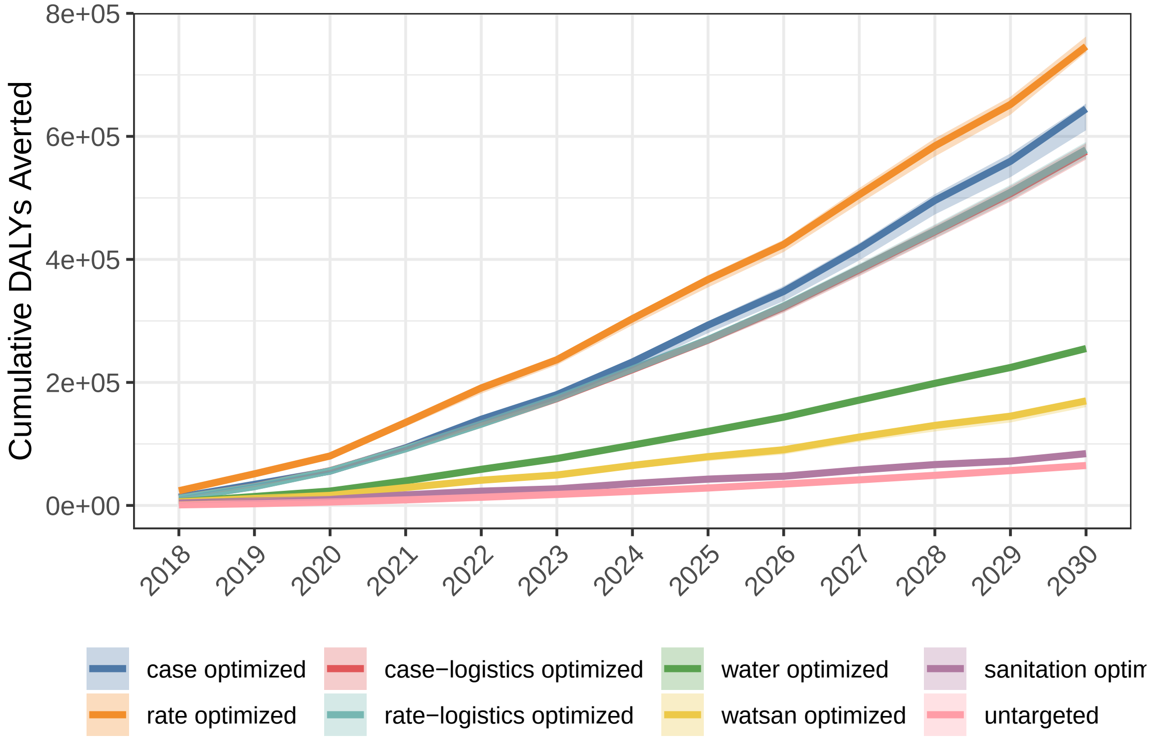

Figure O: **Cumulative DALYs averted from mass oral cholera vaccination campaigns across all vaccination deployment strategies in sub-Saharan Africa from 2018 through 2030.**

Summary Tables by Parameter Set

This section presents summary tables of model outcomes by sensitivity parameter set.

Baseline

Table D: **Summary of cumulative health impacts and total cost per DALY averted (USD 2017) from 2018-2030 by vaccination deployment strategy for baseline model parameters.**

Deployment Strategy	Measure	Mean	2.5%	97.5%
case-logistics optimized	cases averted	616188	597745	642740
case optimized	cases averted	693007	648877	722717
rate-logistics optimized	cases averted	617424	599150	643791
rate optimized	cases averted	828971	803370	859980
sanitation optimized	cases averted	109817	103735	114110
untargeted	cases averted	66562	65733	67702
water optimized	cases averted	273939	270319	277002
watsan optimized	cases averted	197714	187610	203934
case-logistics optimized	deaths averted	24104	23501	25039
case optimized	deaths averted	27192	25980	28116
rate-logistics optimized	deaths averted	24189	23579	25115
rate optimized	deaths averted	31958	31503	33011
sanitation optimized	deaths averted	3682	3469	3812
untargeted	deaths averted	2771	2745	2813
water optimized	deaths averted	10672	10517	10827
watsan optimized	deaths averted	7144	6760	7401
case-logistics optimized	DALYs averted	575078	562373	588721
case optimized	DALYs averted	637975	609931	653243
rate-logistics optimized	DALYs averted	577533	564572	590935
rate optimized	DALYs averted	746749	736607	762273
sanitation optimized	DALYs averted	83228	78579	86117
untargeted	DALYs averted	64862	64404	65532
water optimized	DALYs averted	255090	251723	258787
watsan optimized	DALYs averted	168663	159833	174676
case-logistics optimized	% cases averted	25	25	26
case optimized	% cases averted	28	27	29
rate-logistics optimized	% cases averted	25	25	26
rate optimized	% cases averted	34	33	35
sanitation optimized	% cases averted	5	4	5
untargeted	% cases averted	3	3	3
water optimized	% cases averted	11	11	11
watsan optimized	% cases averted	8	8	8
case-logistics optimized	cost per DALY averted	2393	1332	3116
case optimized	cost per DALY averted	2158	1199	2878
rate-logistics optimized	cost per DALY averted	2383	1327	3102
rate optimized	cost per DALY averted	1843	1032	2382
sanitation optimized	cost per DALY averted	16546	9121	22243
untargeted	cost per DALY averted	21213	11953	27263
water optimized	cost per DALY averted	5394	3029	6965
watsan optimized	cost per DALY averted	8164	4496	10923

Table E: **Mean percentage of cases averted annually from 2018-2030 by vaccination deployment strategy for baseline model parameters.**

Deployment Strategy	2018	2019	2020	2021	2022	2023	2024	2025	2026	2027	2028	2029	2030
case-logistics optimized	9	14	17	24	26	25	28	29	27	30	30	29	31
case optimized	10	15	15	24	31	26	31	36	29	34	39	31	37
rate-logistics optimized	9	14	17	24	26	26	29	28	28	30	30	29	31
rate optimized	21	21	20	39	37	27	43	38	28	43	40	31	47
sanitation optimized	3	3	2	6	4	3	6	5	3	7	6	3	7
untargeted	1	1	2	2	3	3	3	3	3	3	3	4	4
water optimized	6	7	7	11	12	11	13	13	11	14	13	12	14
watsan optimized	5	5	4	9	8	6	10	9	6	11	10	7	12

Vaccine Efficacy

Table F: **Summary of cumulative health impacts and total cost per DALY averted from 2018-2030 by vaccination deployment strategy for low vaccine efficacy model parameters.**

Deployment Strategy	Measure	Mean	2.5%	97.5%
case-logistics optimized	cases averted	326515	316982	340464
case optimized	cases averted	369137	345440	384869
rate-logistics optimized	cases averted	327201	317673	341095
rate optimized	cases averted	438899	426065	454912
sanitation optimized	cases averted	58367	55054	60609
untargeted	cases averted	44065	43516	44820
water optimized	cases averted	144668	142815	146277
watsan optimized	cases averted	105076	99749	108439
case-logistics optimized	deaths averted	12788	12473	13283
case optimized	deaths averted	14486	13831	14965
rate-logistics optimized	deaths averted	12836	12516	13325
rate optimized	deaths averted	16931	16702	17470
sanitation optimized	deaths averted	1948	1835	2017
untargeted	deaths averted	1835	1817	1862
water optimized	deaths averted	5643	5562	5724
watsan optimized	deaths averted	3809	3606	3947
case-logistics optimized	DALYs averted	303989	297462	311136
case optimized	DALYs averted	339044	323911	347065
rate-logistics optimized	DALYs averted	305332	298553	312372
rate optimized	DALYs averted	394463	389749	402247
sanitation optimized	DALYs averted	44009	41524	45542
untargeted	DALYs averted	42856	42553	43297
water optimized	DALYs averted	133941	132192	135859
watsan optimized	DALYs averted	89666	85059	92907
case-logistics optimized	% cases averted	13	13	14
case optimized	% cases averted	15	14	16
rate-logistics optimized	% cases averted	13	13	14
rate optimized	% cases averted	18	18	18
sanitation optimized	% cases averted	2	2	2
untargeted	% cases averted	2	2	2
water optimized	% cases averted	6	6	6
watsan optimized	% cases averted	4	4	4
case-logistics optimized	cost per DALY averted	4527	2521	5890
case optimized	cost per DALY averted	4061	2257	5419
rate-logistics optimized	cost per DALY averted	4507	2512	5864
rate optimized	cost per DALY averted	3488	1955	4500
sanitation optimized	cost per DALY averted	31290	17256	42083
untargeted	cost per DALY averted	32106	18091	41263
water optimized	cost per DALY averted	10273	5771	13261
watsan optimized	cost per DALY averted	15356	8451	20533

Table G: **Summary of cumulative health impacts and total cost per DALY averted from 2018-2030 by vaccination deployment strategy for high vaccine efficacy model parameters.**

Deployment Strategy	Measure	Mean	2.5%	97.5%
case-logistics optimized	cases averted	776640	753350	810300
case optimized	cases averted	874109	818049	912103
rate-logistics optimized	cases averted	778189	755252	811747
rate optimized	cases averted	1041303	1009714	1079703
sanitation optimized	cases averted	137740	129874	143224
untargeted	cases averted	87228	86142	88722
water optimized	cases averted	344465	339953	348321
watsan optimized	cases averted	247919	235089	255750
case-logistics optimized	deaths averted	30383	29628	31572
case optimized	deaths averted	34282	32744	35456
rate-logistics optimized	deaths averted	30492	29723	31670
rate optimized	deaths averted	40175	39628	41474
sanitation optimized	deaths averted	4615	4344	4779
untargeted	deaths averted	3632	3598	3687
water optimized	deaths averted	13428	13233	13622
watsan optimized	deaths averted	8959	8471	9283
case-logistics optimized	DALYs averted	724350	708430	741730
case optimized	DALYs averted	803911	768384	823453
rate-logistics optimized	DALYs averted	727449	711170	744616
rate optimized	DALYs averted	938353	926143	957303
sanitation optimized	DALYs averted	104299	98360	107962
untargeted	DALYs averted	85100	84499	85979
water optimized	DALYs averted	320492	316223	325113
watsan optimized	DALYs averted	211291	200049	218887
case-logistics optimized	% cases averted	32	31	33
case optimized	% cases averted	36	34	37
rate-logistics optimized	% cases averted	32	31	33
rate optimized	% cases averted	43	42	44
sanitation optimized	% cases averted	6	5	6
untargeted	% cases averted	4	4	4
water optimized	% cases averted	14	14	14
watsan optimized	% cases averted	10	10	10
case-logistics optimized	cost per DALY averted	1900	1057	2474
case optimized	cost per DALY averted	1713	951	2284
rate-logistics optimized	cost per DALY averted	1892	1053	2463
rate optimized	cost per DALY averted	1466	821	1894
sanitation optimized	cost per DALY averted	13203	7277	17774
untargeted	cost per DALY averted	16169	9110	20780
water optimized	cost per DALY averted	4293	2412	5543
watsan optimized	cost per DALY averted	6517	3588	8727

Indirect Vaccine Protection

Table H: **Summary of cumulative health impacts and total cost per DALY averted from 2018-2030 by vaccination deployment strategy for a model with no indirect effects.**

Deployment Strategy	Measure	Mean	2.5%	97.5%
case-logistics optimized	cases averted	393974	382279	410984
case optimized	cases averted	444490	416182	463785
rate-logistics optimized	cases averted	394674	383061	411605
rate optimized	cases averted	529020	512701	548680
sanitation optimized	cases averted	70144	66157	72894
untargeted	cases averted	66066	65243	67197
water optimized	cases averted	174812	172542	176762
watsan optimized	cases averted	126317	119763	130363
case-logistics optimized	deaths averted	15414	15026	16017
case optimized	deaths averted	17433	16659	18032
rate-logistics optimized	deaths averted	15466	15074	16062
rate optimized	deaths averted	20399	20106	21066
sanitation optimized	deaths averted	2347	2210	2430
untargeted	deaths averted	2751	2725	2792
water optimized	deaths averted	6813	6715	6911
watsan optimized	deaths averted	4569	4319	4736
case-logistics optimized	DALYs averted	367147	359046	375900
case optimized	DALYs averted	408695	390801	418639
rate-logistics optimized	DALYs averted	368626	360381	377295
rate optimized	DALYs averted	476104	469564	485864
sanitation optimized	DALYs averted	53035	50036	54897
untargeted	DALYs averted	64381	63927	65046
water optimized	DALYs averted	162335	160188	164661
watsan optimized	DALYs averted	107688	101966	111589
case-logistics optimized	% cases averted	16	16	17
case optimized	% cases averted	18	17	19
rate-logistics optimized	% cases averted	16	16	17
rate optimized	% cases averted	22	21	22
sanitation optimized	% cases averted	3	3	3
untargeted	% cases averted	3	3	3
water optimized	% cases averted	7	7	7
watsan optimized	% cases averted	5	5	5
case-logistics optimized	cost per DALY averted	3748	2086	4880
case optimized	cost per DALY averted	3369	1871	4492
rate-logistics optimized	cost per DALY averted	3733	2079	4859
rate optimized	cost per DALY averted	2890	1618	3736
sanitation optimized	cost per DALY averted	25966	14315	34927
untargeted	cost per DALY averted	21372	12042	27467
water optimized	cost per DALY averted	8476	4762	10943
watsan optimized	cost per DALY averted	12786	7038	17125

Table I: **Summary of cumulative health impacts and total cost per DALY averted from 2018-2030 by vaccination deployment strategy for high indirect effects model parameters.**

Deployment Strategy	Measure	Mean	2.5%	97.5%
case-logistics optimized	cases averted	851603	826476	888509
case optimized	cases averted	963346	901803	1005654
rate-logistics optimized	cases averted	853018	828029	889882
rate optimized	cases averted	1141545	1106561	1183582
sanitation optimized	cases averted	151537	142706	157630
untargeted	cases averted	630214	622362	641010
water optimized	cases averted	377128	372274	381323
watsan optimized	cases averted	272743	258422	281626
case-logistics optimized	deaths averted	33324	32487	34639
case optimized	deaths averted	37770	36091	39081
rate-logistics optimized	deaths averted	33436	32589	34735
rate optimized	deaths averted	44032	43408	45455
sanitation optimized	deaths averted	5063	4765	5243
untargeted	deaths averted	26239	25993	26637
water optimized	deaths averted	14706	14495	14914
watsan optimized	deaths averted	9872	9326	10236
case-logistics optimized	DALYs averted	792707	775300	811740
case optimized	DALYs averted	884863	846119	906590
rate-logistics optimized	DALYs averted	795833	777984	814787
rate optimized	DALYs averted	1026750	1012775	1047432
sanitation optimized	DALYs averted	114361	107816	118411
untargeted	DALYs averted	608387	604096	614560
water optimized	DALYs averted	349479	344863	354419
watsan optimized	DALYs averted	232364	219850	240889
case-logistics optimized	% cases averted	35	34	36
case optimized	% cases averted	40	37	41
rate-logistics optimized	% cases averted	35	34	36
rate optimized	% cases averted	47	46	48
sanitation optimized	% cases averted	6	6	6
untargeted	% cases averted	26	26	26
water optimized	% cases averted	15	15	16
watsan optimized	% cases averted	11	11	11
case-logistics optimized	cost per DALY averted	1736	966	2260
case optimized	cost per DALY averted	1556	864	2075
rate-logistics optimized	cost per DALY averted	1729	963	2250
rate optimized	cost per DALY averted	1340	750	1732
sanitation optimized	cost per DALY averted	12042	6637	16209
untargeted	cost per DALY averted	2262	1275	2907
water optimized	cost per DALY averted	3937	2212	5082
watsan optimized	cost per DALY averted	5926	3260	7943

Campaign Frequency

Table J: **Summary of cumulative health impacts and total cost per DALY averted from 2018-2030 by vaccination deployment strategy for the low campaign frequency (every 5 years) parameter set.**

Deployment Strategy	Measure	Mean	2.5%	97.5%
case-logistics optimized	cases averted	414603	399415	425447
case optimized	cases averted	499738	491281	509616
rate-logistics optimized	cases averted	414705	399255	425104
rate optimized	cases averted	568645	561335	578023
sanitation optimized	cases averted	88174	83557	91277
untargeted	cases averted	66562	65733	67702
water optimized	cases averted	197050	195011	200476
watsan optimized	cases averted	142130	136703	146000
case-logistics optimized	deaths averted	16553	15891	17589
case optimized	deaths averted	20134	19256	21169
rate-logistics optimized	deaths averted	16566	15885	17574
rate optimized	deaths averted	22703	21706	23625
sanitation optimized	deaths averted	2993	2838	3104
untargeted	deaths averted	2771	2745	2813
water optimized	deaths averted	7740	7654	7872
watsan optimized	deaths averted	5153	4948	5307
case-logistics optimized	DALYs averted	388991	375058	410773
case optimized	DALYs averted	469219	450203	491873
rate-logistics optimized	DALYs averted	389509	375104	410722
rate optimized	DALYs averted	528588	510182	548806
sanitation optimized	DALYs averted	70269	66677	72882
untargeted	DALYs averted	64862	64404	65532
water optimized	DALYs averted	183303	181155	186502
watsan optimized	DALYs averted	121593	117068	125017
case-logistics optimized	% cases averted	17	17	18
case optimized	% cases averted	21	20	21
rate-logistics optimized	% cases averted	17	17	18
rate optimized	% cases averted	23	23	24
sanitation optimized	% cases averted	4	3	4
untargeted	% cases averted	3	3	3
water optimized	% cases averted	8	8	8
watsan optimized	% cases averted	6	6	6
case-logistics optimized	cost per DALY averted	3540	1906	4668
case optimized	cost per DALY averted	2935	1593	3880
rate-logistics optimized	cost per DALY averted	3536	1906	4665
rate optimized	cost per DALY averted	2605	1427	3438
sanitation optimized	cost per DALY averted	19596	10780	26247
untargeted	cost per DALY averted	21213	11953	27263
water optimized	cost per DALY averted	7507	4207	9679
watsan optimized	cost per DALY averted	11320	6279	14954

Table K: **Summary of cumulative health impacts and total cost per DALY averted from 2018-2030 by vaccination deployment strategy for the high campaign frequency (every 2 years) parameter set.**

Deployment Strategy	Measure	Mean	2.5%	97.5%
case-logistics optimized	cases averted	733941	706351	768438
case optimized	cases averted	746159	705970	785180
rate-logistics optimized	cases averted	733837	706125	768390
rate optimized	cases averted	1045568	992102	1093351
sanitation optimized	cases averted	133530	124489	139523
untargeted	cases averted	66562	65733	67702
water optimized	cases averted	328983	322016	335705
watsan optimized	cases averted	228621	212604	238137
case-logistics optimized	deaths averted	28559	27680	29796
case optimized	deaths averted	29064	27866	30523
rate-logistics optimized	deaths averted	28573	27689	29815
rate optimized	deaths averted	39713	38256	41442
sanitation optimized	deaths averted	4576	4264	4784
untargeted	deaths averted	2771	2745	2813
water optimized	deaths averted	12323	12045	12584
watsan optimized	deaths averted	7992	7454	8297
case-logistics optimized	DALYs averted	687060	666820	706338
case optimized	DALYs averted	679724	653678	701939
rate-logistics optimized	DALYs averted	687962	667609	707329
rate optimized	DALYs averted	924602	890844	951704
sanitation optimized	DALYs averted	102401	95531	106982
untargeted	DALYs averted	64862	64404	65532
water optimized	DALYs averted	294641	288776	299894
watsan optimized	DALYs averted	185382	173188	192427
case-logistics optimized	% cases averted	30	29	31
case optimized	% cases averted	31	29	32
rate-logistics optimized	% cases averted	30	29	31
rate optimized	% cases averted	43	41	44
sanitation optimized	% cases averted	5	5	6
untargeted	% cases averted	3	3	3
water optimized	% cases averted	14	13	14
watsan optimized	% cases averted	9	9	10
case-logistics optimized	cost per DALY averted	2003	1111	2629
case optimized	cost per DALY averted	2025	1119	2681
rate-logistics optimized	cost per DALY averted	2001	1109	2626
rate optimized	cost per DALY averted	1489	826	1969
sanitation optimized	cost per DALY averted	13453	7356	18280
untargeted	cost per DALY averted	21213	11953	27263
water optimized	cost per DALY averted	4670	2611	6066
watsan optimized	cost per DALY averted	7429	4074	10072

Campaign Coverage

Table L: **Summary of cumulative health impacts and total cost per DALY averted from 2018-2030 by vaccination deployment strategy for low vaccination coverage model parameters.**

Deployment Strategy	Measure	Mean	2.5%	97.5%
case-logistics optimized	cases averted	418326	407178	431672
case optimized	cases averted	522825	500475	535522
rate-logistics optimized	cases averted	419621	408671	433002
rate optimized	cases averted	583203	576147	592731
sanitation optimized	cases averted	83844	78774	86877
untargeted	cases averted	66562	65733	67702
water optimized	cases averted	191337	189535	193777
watsan optimized	cases averted	142673	138285	147706
case-logistics optimized	deaths averted	16640	16227	17033
case optimized	deaths averted	20916	20272	22079
rate-logistics optimized	deaths averted	16683	16273	17075
rate optimized	deaths averted	23100	22143	23884
sanitation optimized	deaths averted	2738	2552	2843
untargeted	deaths averted	2771	2745	2813
water optimized	deaths averted	7516	7433	7613
watsan optimized	deaths averted	5267	5069	5456
case-logistics optimized	DALYs averted	394412	384971	401189
case optimized	DALYs averted	489638	476652	515578
rate-logistics optimized	DALYs averted	395410	386278	402003
rate optimized	DALYs averted	540918	523208	558450
sanitation optimized	DALYs averted	63536	59138	65959
untargeted	DALYs averted	64862	64404	65532
water optimized	DALYs averted	179331	177509	181684
watsan optimized	DALYs averted	124614	120135	128890
case-logistics optimized	% cases averted	17	17	17
case optimized	% cases averted	21	21	22
rate-logistics optimized	% cases averted	17	17	18
rate optimized	% cases averted	24	24	24
sanitation optimized	% cases averted	3	3	4
untargeted	% cases averted	3	3	3
water optimized	% cases averted	8	8	8
watsan optimized	% cases averted	6	6	6
case-logistics optimized	cost per DALY averted	7757	1952	20935
case optimized	cost per DALY averted	6252	1522	16940
rate-logistics optimized	cost per DALY averted	7768	1947	20881
rate optimized	cost per DALY averted	5674	1405	15476
sanitation optimized	cost per DALY averted	48636	11924	136337
untargeted	cost per DALY averted	47196	11962	125964
water optimized	cost per DALY averted	17006	4316	45615
watsan optimized	cost per DALY averted	24453	6102	67274

Table M: **Summary of cumulative health impacts and total cost per DALY averted from 2018-2030 by vaccination deployment strategy for high vaccination coverage model parameters.**

Deployment Strategy	Measure	Mean	2.5%	97.5%
case-logistics optimized	cases averted	691262	666546	723443
case optimized	cases averted	732105	688897	768931
rate-logistics optimized	cases averted	698111	673333	730590
rate optimized	cases averted	953179	911923	994805
sanitation optimized	cases averted	119392	112105	124480
untargeted	cases averted	66562	65733	67702
water optimized	cases averted	309335	303988	313822
watsan optimized	cases averted	208116	195495	214804
case-logistics optimized	deaths averted	26869	26114	27985
case optimized	deaths averted	28568	27360	29859
rate-logistics optimized	deaths averted	27177	26406	28321
rate optimized	deaths averted	36479	35418	37975
sanitation optimized	deaths averted	4102	3855	4279
untargeted	deaths averted	2771	2745	2813
water optimized	deaths averted	11796	11568	12013
watsan optimized	deaths averted	7332	6865	7574
case-logistics optimized	DALYs averted	644035	626957	661557
case optimized	DALYs averted	669097	641934	689465
rate-logistics optimized	DALYs averted	651998	634594	670096
rate optimized	DALYs averted	848872	824779	872312
sanitation optimized	DALYs averted	92182	86766	96086
untargeted	DALYs averted	64862	64404	65532
water optimized	DALYs averted	280290	275552	284962
watsan optimized	DALYs averted	170781	159894	176213
case-logistics optimized	% cases averted	28	27	29
case optimized	% cases averted	30	28	31
rate-logistics optimized	% cases averted	29	28	30
rate optimized	% cases averted	39	38	40
sanitation optimized	% cases averted	5	5	5
untargeted	% cases averted	3	3	3
water optimized	% cases averted	13	12	13
watsan optimized	% cases averted	9	8	9
case-logistics optimized	cost per DALY averted	4752	1187	12914
case optimized	cost per DALY averted	4575	1137	12622
rate-logistics optimized	cost per DALY averted	4712	1172	12753
rate optimized	cost per DALY averted	3615	904	9825
sanitation optimized	cost per DALY averted	33508	8205	92816
untargeted	cost per DALY averted	47196	11962	125964
water optimized	cost per DALY averted	10881	2749	29385
watsan optimized	cost per DALY averted	17850	4455	50172

Vaccine Supply

Table N: **Summary of cumulative health impacts and total cost per DALY averted from 2018-2030 by vaccination deployment strategy for the low vaccine supply (up to 26 million doses in 2030) parameter set.**

Deployment Strategy	Measure	Mean	2.5%	97.5%
case-logistics optimized	cases averted	467436	452372	487583
case optimized	cases averted	475185	448519	503639
rate-logistics optimized	cases averted	485513	468662	505693
rate optimized	cases averted	710856	670679	744365
sanitation optimized	cases averted	89180	83697	92791
untargeted	cases averted	44162	43613	44918
water optimized	cases averted	224957	219843	229532
watsan optimized	cases averted	151121	140798	157186
case-logistics optimized	deaths averted	18191	17686	18926
case optimized	deaths averted	18496	17700	19560
rate-logistics optimized	deaths averted	19043	18483	19782
rate optimized	deaths averted	26866	25753	28116
sanitation optimized	deaths averted	3053	2866	3176
untargeted	deaths averted	1839	1822	1867
water optimized	deaths averted	8395	8183	8594
watsan optimized	deaths averted	5280	4930	5478
case-logistics optimized	DALYs averted	434799	423910	445718
case optimized	DALYs averted	426640	409677	443534
rate-logistics optimized	DALYs averted	456382	443814	468410
rate optimized	DALYs averted	620776	595037	640358
sanitation optimized	DALYs averted	67935	63852	70647
untargeted	DALYs averted	42634	42335	43064
water optimized	DALYs averted	198652	194172	202740
watsan optimized	DALYs averted	121434	113464	125924
case-logistics optimized	% cases averted	19	19	20
case optimized	% cases averted	20	19	20
rate-logistics optimized	% cases averted	20	19	20
rate optimized	% cases averted	29	28	30
sanitation optimized	% cases averted	4	3	4
untargeted	% cases averted	2	2	2
water optimized	% cases averted	9	9	9
watsan optimized	% cases averted	6	6	6
case-logistics optimized	cost per DALY averted	2048	1137	2678
case optimized	cost per DALY averted	2088	1148	2766
rate-logistics optimized	cost per DALY averted	1951	1082	2557
rate optimized	cost per DALY averted	1435	794	1908
sanitation optimized	cost per DALY averted	13117	7209	17685
untargeted	cost per DALY averted	20881	11768	26836
water optimized	cost per DALY averted	4482	2500	5840
watsan optimized	cost per DALY averted	7338	4029	9954

Table O: **Summary of cumulative health impacts and total cost per DALY averted from 2018-2030 by vaccination deployment strategy for the high vaccine supply (up to 95 million doses in 2030) parameter set.**

Deployment Strategy	Measure	Mean	2.5%	97.5%
case-logistics optimized	cases averted	683745	662012	702958
case optimized	cases averted	827238	801111	846488
rate-logistics optimized	cases averted	683541	661693	702757
rate optimized	cases averted	951412	940803	966999
sanitation optimized	cases averted	143553	135622	148327
untargeted	cases averted	92891	91733	94483
water optimized	cases averted	334830	331615	339587
watsan optimized	cases averted	233431	224152	240073
case-logistics optimized	deaths averted	27216	26333	28244
case optimized	deaths averted	33204	32065	35005
rate-logistics optimized	deaths averted	27195	26306	28222
rate optimized	deaths averted	37772	36251	38978
sanitation optimized	deaths averted	4815	4540	4990
untargeted	deaths averted	3867	3831	3926
water optimized	deaths averted	13205	13064	13400
watsan optimized	deaths averted	8484	8143	8749
case-logistics optimized	DALYs averted	645756	626824	667274
case optimized	DALYs averted	779348	755177	819210
rate-logistics optimized	DALYs averted	645458	626468	666987
rate optimized	DALYs averted	885827	857653	911128
sanitation optimized	DALYs averted	113092	106699	117278
untargeted	DALYs averted	90913	90269	91861
water optimized	DALYs averted	317400	313809	322136
watsan optimized	DALYs averted	201756	194159	207630
case-logistics optimized	% cases averted	28	27	29
case optimized	% cases averted	34	33	35
rate-logistics optimized	% cases averted	28	27	29
rate optimized	% cases averted	39	39	40
sanitation optimized	% cases averted	6	6	6
untargeted	% cases averted	4	4	4
water optimized	% cases averted	14	14	14
watsan optimized	% cases averted	10	9	10
case-logistics optimized	cost per DALY averted	3016	1659	3950
case optimized	cost per DALY averted	2500	1353	3279
rate-logistics optimized	cost per DALY averted	3017	1660	3952
rate optimized	cost per DALY averted	2199	1216	2893
sanitation optimized	cost per DALY averted	17230	9477	23202
untargeted	cost per DALY averted	21414	12064	27521
water optimized	cost per DALY averted	6134	3442	7902
watsan optimized	cost per DALY averted	9653	5346	12756

Population Turnover

Table P: **Summary of cumulative health impacts and total cost per DALY averted from 2018-2030 by vaccination deployment strategy for the high population turnover rate/low life expectancy (56 years) parameter set.**

Deployment Strategy	Measure	Mean	2.5%	97.5%
case-logistics optimized	cases averted	613686	595328	640112
case optimized	cases averted	690216	646263	719790
rate-logistics optimized	cases averted	614866	596690	641100
rate optimized	cases averted	825662	800179	856550
sanitation optimized	cases averted	109391	103335	113665
untargeted	cases averted	66347	65520	67483
water optimized	cases averted	272826	269222	275877
watsan optimized	cases averted	196941	186880	203137
case-logistics optimized	deaths averted	24006	23406	24937
case optimized	deaths averted	27083	25876	28002
rate-logistics optimized	deaths averted	24089	23482	25009
rate optimized	deaths averted	31830	31378	32879
sanitation optimized	deaths averted	3668	3455	3797
untargeted	deaths averted	2762	2737	2804
water optimized	deaths averted	10629	10474	10783
watsan optimized	deaths averted	7116	6734	7372
case-logistics optimized	DALYs averted	572737	560091	586310
case optimized	DALYs averted	635405	607466	650599
rate-logistics optimized	DALYs averted	575106	562227	588428
rate optimized	DALYs averted	743747	733673	759205
sanitation optimized	DALYs averted	82903	78273	85780
untargeted	DALYs averted	64652	64195	65319
water optimized	DALYs averted	254045	250689	257727
watsan optimized	DALYs averted	168007	159217	173997
case-logistics optimized	% cases averted	25	25	26
case optimized	% cases averted	28	27	29
rate-logistics optimized	% cases averted	25	25	26
rate optimized	% cases averted	34	33	35
sanitation optimized	% cases averted	4	4	5
untargeted	% cases averted	3	3	3
water optimized	% cases averted	11	11	11
watsan optimized	% cases averted	8	8	8
case-logistics optimized	cost per DALY averted	2403	1337	3128
case optimized	cost per DALY averted	2167	1204	2889
rate-logistics optimized	cost per DALY averted	2393	1333	3116
rate optimized	cost per DALY averted	1850	1036	2391
sanitation optimized	cost per DALY averted	16610	9157	22330
untargeted	cost per DALY averted	21283	11992	27352
water optimized	cost per DALY averted	5416	3042	6994
watsan optimized	cost per DALY averted	8195	4513	10965

Table Q: **Summary of cumulative health impacts and total cost per DALY averted from 2018-2030 by vaccination deployment strategy for the low population turnover rate/high life expectancy (70 years) parameter set.**

Deployment Strategy	Measure	Mean	2.5%	97.5%
case-logistics optimized	cases averted	683745	662012	702958
case optimized	cases averted	827238	801111	846488
rate-logistics optimized	cases averted	683541	661693	702757
rate optimized	cases averted	951412	940803	966999
sanitation optimized	cases averted	143553	135622	148327
untargeted	cases averted	92891	91733	94483
water optimized	cases averted	334830	331615	339587
watsan optimized	cases averted	233431	224152	240073
case-logistics optimized	deaths averted	27216	26333	28244
case optimized	deaths averted	33204	32065	35005
rate-logistics optimized	deaths averted	27195	26306	28222
rate optimized	deaths averted	37772	36251	38978
sanitation optimized	deaths averted	4815	4540	4990
untargeted	deaths averted	3867	3831	3926
water optimized	deaths averted	13205	13064	13400
watsan optimized	deaths averted	8484	8143	8749
case-logistics optimized	DALYs averted	645756	626824	667274
case optimized	DALYs averted	779348	755177	819210
rate-logistics optimized	DALYs averted	645458	626468	666987
rate optimized	DALYs averted	885827	857653	911128
sanitation optimized	DALYs averted	113092	106699	117278
untargeted	DALYs averted	90913	90269	91861
water optimized	DALYs averted	317400	313809	322136
watsan optimized	DALYs averted	201756	194159	207630
case-logistics optimized	% cases averted	28	27	29
case optimized	% cases averted	34	33	35
rate-logistics optimized	% cases averted	28	27	29
rate optimized	% cases averted	39	39	40
sanitation optimized	% cases averted	6	6	6
untargeted	% cases averted	4	4	4
water optimized	% cases averted	14	14	14
watsan optimized	% cases averted	10	9	10
case-logistics optimized	cost per DALY averted	3016	1659	3950
case optimized	cost per DALY averted	2500	1353	3279
rate-logistics optimized	cost per DALY averted	3017	1660	3952
rate optimized	cost per DALY averted	2199	1216	2893
sanitation optimized	cost per DALY averted	17230	9477	23202
untargeted	cost per DALY averted	21414	12064	27521
water optimized	cost per DALY averted	6134	3442	7902
watsan optimized	cost per DALY averted	9653	5346	12756
